# *Halomonas* Rhizobacteria of *Avicennia marina* of Indian Sundarbans Promote Rice Growth Under Saline and Heavy Metal Stresses Through Exopolysaccharide Production

**DOI:** 10.3389/fmicb.2019.01207

**Published:** 2019-05-29

**Authors:** Pritam Mukherjee, Abhijit Mitra, Madhumita Roy

**Affiliations:** ^1^Department of Biotechnology, Techno India University, Kolkata, India; ^2^Department of Marine Science, University of Calcutta, Kolkata, India; ^3^Department of Microbiology, Bose Institute, Kolkata, India

**Keywords:** abiotic stress, arsenic bioremediation, exopolysaccharide, *Halomonas*, rhizobacteria, rice growth promotion, salt sequestration, true mangrove *Avicennia marina*

## Abstract

The *Halomonas* species isolated from the rhizosphere of the true mangrove *Avicennia marina* of Indian Sundarbans showed enhanced rice growth promotion under combined stress of salt and arsenic in pot assay. Interestingly, under abiotic stress conditions, *Halomonas* sp. Exo1 was observed as an efficient producer of exopolysaccharide. The study revealed that salt triggered exopolysaccharide production, which in turn, increased osmotic tolerance of the strain. Again, like salt, presence of arsenic also caused increased exopolysaccharide production that in turn sequestered arsenic showing a positive feedback mechanism. To understand the role of exopolysaccharide in salt and arsenic biosorption, purified exopolysaccharide mediated salt and arsenic sequestration were studied both under *in vivo* and *in vitro* conditions and the substrate binding properties were characterized through FT-IR and SEM-EDX analyses. Finally, observation of enhanced plant growth in pot assay in the presence of the strain and pure exopolysaccharide separately, confirmed direct role of exopolysaccharide in plant growth promotion.

## Introduction

Soil salinity is one of the major abiotic stresses that limit plant productivity throughout the globe. Increased salinity due to climate change and sea level rise is causing daily extinction of many flora and fauna in the coastal world including Indian Sundarbans, inscribed as a World Heritage site by UNESCO (Mitra and Zaman, 2016). In Indian Sundarbans, rice cultivation (80–90% of total crop production in India) is the primary agricultural activity and is the main source of income generation but is restricted mainly to the monsoon season due to high salinity of soil in other seasons (Yadav et al., 1979). In addition to the existing problem of soil salinity, toxic heavy metal (HM) contamination in Indian Sundarbans is another major abiotic stress (Pal et al., 2002). Heavy metal pollution has attained a serious threat here due to discharge of HM rich semi-treated or untreated effluents from industries (tanneries, jute mills, pulp and paper mills, pesticide manufacturing plants, thermal power plants, brick kilns, rubber, fertilizer and soap factories, antibiotic plants, and oil refineries), located on the banks of the river Hooghly. Slow continuous release of HM from the antifouling paints used to coat the bottom of the numerous fishing trawlers and recreational vessels is another reason of HM pollution in this region (Bighiu et al., 2017). Upon entering the estuarine system, these HMs get dissolubilized or precipitated on the soil and sediment based on the localized environmental conditions especially pH. Nevertheless, over the years, the region has turned out as a natural sink by virtue of its rich and unique indigenous flora, fauna and a diverse group of largely unexplored mangrove microbiota (Mitra, 2013). Previous research on Indian saline ecosystem identified the potential of eco-friendly halo-tolerant plant growth-promoting rhizomicrobes (PGPR) for application in sustenance of agronomy under diverse saline ecologies (Shrivastava and Kumar, 2015). Application of these native PGPR to alleviate salt stress can be regarded as a better alternative toward sustainable agriculture than developing salt and other stress tolerant transgenic varieties due to high production cost and other environmental hazards (Shrivastava and Kumar, 2015). These osmotolerant microbes possess special mechanisms such as optimization of Na^+^/K^+^ flow, accumulation of essential osmolytes, transcriptional, and/or translational regulation of salt-tolerant enzymes (osmozymes) for sustaining environmental osmotic shock (Yang et al., 2009). In comparison to the mesophilic (halo-sensitive) counterparts, halo-tolerant microbes are more dynamic owing to their ability to function under both stress and stress-free conditions (Nadeem et al., 2014). Yang et al. (2009) pointed out that the halo-tolerant PGPR causes “induced systemic tolerance” (IST) to salt-sensitive plants and enhances nutrient uptake from soils, thus decreasing the need of the fertilizers. In general, salt-sensitive plants cannot grow or show poor growth in saline media due to ion toxicity (replacement of K^+^ by Na^+^ in biochemical reactions, and Na^+^ and Cl^−^ induced conformational changes in proteins), osmotic stress (excessive accumulation of sodium in cell walls causing osmotic stress and cell death), nutrient (N, Ca, K, P, Fe, Zn) deficiency due to salt related imbalance and oxidative stress on plants apart from reduced water uptake from the soil. On the other hand, in salt-tolerant plants, salt stress activates specialized genes that are involved in the sustenance of salt-induced and other abiotic stresses (Yang et al., 2009). As salt-sensitive plants do not possess these special genes, their salt stress is ameliorated by the PGPR mediated IST. Although bacterial determinants of IST and their activation pathways in plants have been significantly evaluated during the past decade but the response of the PGPR toward the abiotic stresses has been revealed very recently (Timmusk and Wagner, 1999; Zhang et al., 2008). Use of these PGPR for minimizing agricultural chemical load, HM load (Nadeem et al., 2014; Yu et al., 2014) and soil salinity (Siddikee et al., 2010) is earning considerable appreciation in boosting environmental quality. Although salt stress alleviation by PGPR inoculants had already been reported in rice, wheat and many other crops (Goswami et al., 2014), but the exact mechanism of salt stress alleviation is not fully explored. In addition, exopolysaccharide (EPS) production was noticed in a number of halophilic bacteria, halophilic PGPR, and metal mobilizing bacteria (Llamas et al., 2006). With a growing interest in extremophiles, a large number of halophiles, particularly species belonging to the *Halomonas* genera have been found to be the possessors of EPS (Béjar et al., 1998; Arias et al., 2003; Martínez-Cánovas et al., 2004; Llamas et al., 2006; Poli et al., 2013).

The purpose of this paper is to understand the role of EPS produced by halophilic PGPR belonging to the genus *Halomonas*, isolated from the rhizosphere of the *Avicennia marina* of Indian Sundarbans, in abiotic stress alleviation during rice growth promotion under combined stress of salt and arsenic (As).

## Materials and Methods

### Sampling and Physico-Chemical Assessment of *Avicennia marina* Rhizosphere Soil

The root associated soil of *Avicennia marina* seedling growing in the intertidal mud flat of Bonnie Camp (21°49′45.3″ N; 88°37′13.6″ E) situated in the central region of Indian Sundarbans was collected during monsoon season in the month of September, 2014. The sample was sieved (pore size of 2 mm), grounded to powder using a mortar and pestle, and stored in a clean polyethylene bag at 4°C until use. The soil salinity, electrical conductivity, and pH were recorded with refractometer (Atago, Tokyo, Japan), digital electrical conductivity/TDS meter (Global, India) and digital pH meter (Global, India), respectively, on spot. Moisture content was measured in lab by subtracting dry weight (in oven at 105°C) from the wet weight of the sample. Soil chemical features like Soil Organic Carbon (SOC), % organic matter, N, P, K (U.S. Environmental Protection Agency, 1994) and biologically available HMs were determined (Malo, 1977).

### Isolation and Identification of Exopolysaccharide Producing Arsenic and Salt Resistant Halo-Rhizobacteria

Enrichment and isolation of halo-rhizobacteria from *A. marina* rhizosphere soil was done in 8% total salt modified growth media (8% TSMGM) [a specific medium for cultivation of halophiles and haloarchaea that contain 266.7 ml of concentrated salt water (30% stock) {NaCl (240 g l^−1^), MgCl_2_.6H_2_O (30 g l^−1^), MgSO_4_.7H_2_O (35 g l^−1^), KCl (7 g l^−1^), 5 ml 1M Tris-Cl (pH-7.5), 5 ml 1M CaCl_2_.2H_2_O (0.5 g l^−1^), adjusted to pH-7.5 with 1M Tris base}, peptone (5 g l^−1^), yeast extract (1 g l^−1^), adjusted pH to 7.5 with 1M Tris base] (Dyall-Smith, 2009). Isolates that showed moderate growth in 4 mM [As(III)] were selected for further purification. For screening of the EPS secreting isolates, pure colonies were transferred to nutrient agar (NA) media supplemented with 5% dextrose. Isolates showing mucoid phenotypes were finally selected and purified. After morphological study, the isolates were finally identified by 16S rRNA gene analysis using universal PCR primers 27F and 1492R as mentioned by Mukherjee et al. (2017). The amplified products were sequenced according to the manufacturer's specifications for Taq DNA polymerase-initiated cycle sequencing reactions using fluorescently labeled dideoxynucleotide terminators with an ABI PRISM 377 automated sequencer (Perkin-Elmer Applied Biosystems). 16S rRNA gene sequence similarity was determined using BLAST version 2.2.12 of the National Center for Biotechnology Information (Altschul et al., 1990). The partial 16S rRNA gene sequences were submitted to GenBank and accession numbers were obtained. Phylogenetic analyses were performed by neighbor-joining method (Saitou and Nei, 1987) with bootstrapping using 1000 replications using MEGA version 7 (Kumar et al., 2016) with Kimura two-parameter model. The bootstrap consensus tree inferred from 1,000 replicates was taken to represent the evolutionary history of the taxa analyzed. The evolutionary distances were computed using the Maximum Composite Likelihood method (Tamura et al., 2004).

Selected bacterial surface morphology was determined by Field Emission Scanning Electron Microscope (FESEM) (Model JEOLJSM 6700F) after growing the isolate for 5 days in salt media (8% TSMGM) amended with [As(III)]. The bacterial sample was mounted on aluminum stubs with conductive carbon cement, dried for 3 h, and coated with 15 nm platinum film with an agar automatic sputter coater. After processing, the sample was visualized in the high-vacuum mode at 15 kV and the images were processed using Photoshop software (Adobe Systems Inc., Calif.).

### Characterization of the Isolate Toward Abiotic Stress Tolerance: Salt and Heavy Metals

For all growth-related experiments, cultures were set up in triplicates, incubations were performed at 30 ± 2°C for 3–5 days and the growths were determined both visually and spectrophotometrically at OD_600nm_. For determination of the minimal inhibitory concentration (MIC) of the *Halomonas* sp. Exo1 toward total salt and NaCl, agar plates with increasing concentrations of mixed sea salt and NaCl in presence and absence of 2 mM [As(III)] were streaked with pure isolates and incubated. Detailed growth kinetics of the strain was studied in LB with increasing concentrations of NaCl, 8% TSMGM broth, and 8% TSMGM broth with increasing concentrations of total salt and/or [As(III)]. For determination of *in vivo* bioaccumulation of osmolytes including both monovalent (Na^+^, K^+^) and divalent (Mg^2+^, Ca^2+^) cations, 5-day old culture broth was prepared in LB and liquid 8% TSMGM. Centrifugation was done and cell-pellet was washed thrice with deionized H_2_O and oven-dried at 50°C, digested with pure concentrated HNO_3_ (5 ml, Merck) at 65–75°C and filtered through Whatman filter paper. The amounts of osmolytes (Na^+^, K^+^, Mg^2+^, and Ca^2+^) accumulated within cell-biomass were assessed via flame-photometry (Aimil Ltd., India) and the concentrations were calculated from the standard curves of the respective ions. *In vivo* as bioaccumulation study was carried out by live cells and dead cells both. For determination of As accumulation by live cells, time-dependent study was undertaken. The isolate was cultured in 250 ml liquid 8% TSMGM containing 2 mM [As(III)]. 50 ml of culture media was withdrawn at regular intervals of 0, 3, 6, 9, and 12 days. Samples were centrifuged at 7,000 rpm for 10 min to collect both cell-free media and bacterial cell-biomass. Media supernatant was filtered through a 0.22-micron membrane filter (Nuclepore Corp., CA, USA). 10 ml cell-free media filtrate and 0.1 g of dried cell-biomass were digested separately with concentrated HNO_3_ (5 ml, Merck) at 65–75°C on a hot plate equipped with a fume hood and filtered through Whatman filter paper No. 1 (125 mm). Acid-digested media supernatant from As-free salt media (0-day time-point) and acid-digested cell-biomass from As-free salt media (3-day time-point) were used as negative controls. For determination of [As(III)] biosorption by dead halo-rhizobacterial biomass, pellet was obtained from 200 ml 5-day old culture grown in 8% TSMGM in the absence of NaAsO_2_. The cell pellet was washed twice to remove residual media, re-suspended in sterile deionized water, and autoclaved at 121°C at 15 lb/in^2^ for 45 min. After this step, the absence of live bacteria was checked by plating onto 8% TSMGM agar plates. 10 ml dead cell suspension (~7.14 mg bacterial biomass per ml) was incubated at room temperature for 24 h under shaking in the presence of 4 mM [As(III)] and was then centrifuged at 10,000 rpm for 15 min. The cell pellet was again washed thrice with sterile deionized water and dried at 50°C, while the cell-free supernatant was filtered through 0.2–0.45 micron hydrophilic membrane (HiMedia Laboratories Pvt. Ltd.,). Sample preparation and AAS were carried out as mentioned earlier.

Determination of the MIC of the strain for other HMs were done in LB and 8% TSMGM agar plates and broths containing test HMs with increasing concentrations.

*In vivo* as biotransformation assay was done using a qualitative AgNO_3_ screening method as described by Simeonova et al. (2004) with modifications. Briefly, plate-based assay was performed where 8% TSMGM agar plates containing 2 mM [As(III)] were spot inoculated with the test bacterial culture, incubated for 3 days at 30 ± 2°C and flooded with 0.1 M AgNO_3_ solution containing 1 M Tris-HCl (pH 8.0). The reaction between AgNO_3_ and the arsenic species i.e., [As(III)] or [As(V)] in Tris-HCl environment produces colored precipitates containing arsenic. Presence of [As(III)] species by arsenate reducing bacteria produces light yellow color (due to Ag_3_AsO_3_ or silver orthoarsenite) and presence of [As(V)] species by arsenite oxidizing bacteria produces light brownish-red color (due to Ag_3_AsO_4_ or silver orthoarsenate).

### Extraction, Purification, and Estimation of Exopolysaccharide Production Under Salt and Arsenic Stresses

For estimation of EPS production under As stress, 100 μl of 24-h old culture of *Halomonas* sp. Exo1 was inoculated (0.1% v/v) into 250 ml Erlenmeyer flasks containing 100 ml of As-free and As-supplemented (2, 4, 6, 8 mM) liquid 8% TSMGM, mixed thoroughly and placed on a rotary shaker (125 rpm) at 30 ± 2°C for 5 days. For determination of the effect of salt stress on EPS yield of *Halomonas* sp. Exo1, 100 ml Erlenmeyer flasks each containing 50 ml of LB broth with varying concentrations of NaCl (0.5, 1, 2.5, 5, 7.5, 10, 12.5, 15, 20, 22.5 %) were inoculated with the bacterial strain. For extraction of EPS in all the treatments, 5-day old cultures were centrifuged at 7,000 rpm for 20 min. The supernatants were vacuum-filtered through cellulose nitrate filter. The EPS was precipitated from the cell-free supernatant by the addition of ice-cold ethanol (100%, Merck) in 1:3 ratios. The cell-free extract-ethanol mixture was shaken vigorously and incubated overnight at 4°C. For removal of salt and impurities, EPS was dialyzed for 24–48 h using 12–14 kDa MW cut-off dialysis bags against deionized H_2_O at 4°C. A fraction of the partially purified EPS solution was dried at 60°C and weighed to determine crude EPS yield (Corzo et al., 1994). For further purification of EPS, methods described by Bales et al. (2013) were followed. In brief, the EPS solution was mixed with ice-cold 20% (w/v) trichloroacetic acid (TCA) solution to precipitate proteins and nucleic acids. After 30 min of incubation, the solution was centrifuged at 15,000 rpm for 1 h at 4°C. To the supernatant, 1.5 volume of 95% ethanol was added and the mixture was kept at −20°C for 24 h to facilitate precipitation of EPS from lipids. To collect the precipitated EPS, the solution was centrifuged at 15,000 rpm for 1 h at 4°C and the EPS pellet was re-suspended in Milli-Q H_2_O and dialyzed against the same for 24 h at 4°C using a 12–14 kDa MW cut-off dialysis membrane to remove low molecular weight impurities. The remaining retentate containing purified EPS was lyophilized overnight and stored in sterile containers at −20°C until further analyses. The lyophilized powder of purified EPS was weighed again to determine pure EPS yield. In order to extract cell-bound EPS, the bacterial cell-pellet was re-suspended in 300 μl EDTA solution (10 mM EDTA + 1.5 mM NaCl) and heated at 50°C in water bath for 3 min. The bacterial cell suspension was again centrifuged and cell-free supernatant was decanted and EPS was purified as above. Finally, to understand the exact time-point of getting highest EPS yield, total EPS yield was calculated from cultures grown for 0, 3, 6, 9, and 12 days.

### Biophysical Characterization of the Exopolysaccharide

The purified EPS was tested for solubility in various polar and non-polar organic solvents and water. Salinity and electrical conductivity of the pure desalted Exo1 grown in 8% TSMGM were compared with the crude EPS.

Carbohydrate, protein, lipid and sulfate contents of the pure Exo1 were analyzed by spectrophotometer. Total neutral-carbohydrate contents were determined by phenol-sulfuric acid method using D-glucose as a standard (Dubois et al., 1956). The protein contents were measured by Folin-Ciocalteau method using bovine serum albumin (BSA) as a standard (Lowry et al., 1951). Lipid contents were measured by the method described by Novak (Novák, 1965) using olive oil as the standard. Sulfate contents were estimated by barium chloride (BaCl_2_) method using a H_2_SO_4_ standard curve established in the range of 2–20 μg ml^−1^ (Dodgson and Price, 1962). The visual appearance of the purified EPS was evaluated via scanning electron microscopy (SEM). The surface morphology of EPSs was visualized by using SEM (JEOL make JSM6360, UK) with an accelerating voltage of 20 kV (Kavita et al., 2013). Functional groups of crude and purified EPS were determined via Fourier Transformed-Infrared (FT-IR) spectroscopy. The pellets were prepared by grinding 2 mg of EPS with 200 mg of dry potassium bromide (KBr) and the mixture was pressed into a mold of 16 mm diameter. The FT-IR spectra were acquired in the 4,000–400 cm^−1^ region with a resolution of 4 cm^−1^ using an IR- Prestige-21 system (Shimatzu, Japan) (Sardari et al., 2017). Elemental analyses of purified and crude EPS were done using Energy-Dispersive X-ray (EDX) spectroscopy. 5 mg of EPS was attached on a stub and analyzed by SEM-EDX (JEOL make JSM6360, UK). The X-rays emitted by the EPS revealed the weight and atomic percentage of the different elements, which were present in the sample. X-ray diffraction (XRD) analysis was carried out to determine the biophysical properties of the EPS using X-ray diffractometer (RIGAKU make ULTIMA-III, Tokyo, Japan) with Cu target slit (10 mm). The dried EPS sample was examined in powdered form under different operating ranges of 2θ angles between 2 and 80° at a scanning speed of 2° min^−1^ using Cu Kα radiation (λ = 1.54056 Å). The intensity peaks of diffracted X-rays were recorded continuously and the d-spacings corresponding to diffracted X-rays at that value of “θ” were calculated using Bragg's law(D = λ ÷ 2 Sinθ where “θ” is half of the scattering angle measured from the incident beam) (Ricou et al., 2005).

### Functional Characterization of the Exopolysaccharide

Purified Exo1 was examined for *in vivo* and *in vitro* As and salt sequestration/biosorption. Determination of *in vivo* bioadsorption of As by Exo1 was done using both concentration-dependent and time-dependent assays. *Halomonas* sp. Exo1 was cultured in 250 ml liquid 8% TSMGM containing either 0, 2, 4, 6 mM As for 3 days (concentration-dependent) or 2 mM As for 3, 6, 9, and 12 days (time-dependent) at 30 ± 2°C under constant agitation. At each time-point, cell-secreted EPS was extracted from each 100 ml cell-free extract (0, 2, 4, 6 mM As) as described earlier. 0.5 g of oven-dried bacterial EPS was transferred to a 100 ml beaker containing 10 ml of deionized H_2_O and 5 ml of concentrated HNO_3_ (Merck), and digested overnight at 65–75°C followed by filtration using a Whatman filter paper (125 mm) in a volumetric flask. Acid digested EPS from 3-day (As-minus) time-point was used as a negative control for both concentration-dependent and time-dependent assays. Detection of EPS-bound As was carried out using AAS (novAA 350, Analytik Jena) as per standard protocol stated in APHA (2012). On the other hand, *in vitro* bioadsorption of [As(III)] and [As(V)] or metal binding by purified Exo1 was determined by methods of Bhaskar and Bhosle (2006) with modifications. Briefly, bacterial EPS was extracted from liquid 8% TSMGM in the absence of As and 1 g dried EPS was dissolved in 10 ml Milli-Q H_2_O and 2 ml of this EPS solution was taken in a dialysis bag (12–14 kDa MW cut-off, HiMedia Laboratories Pvt. Ltd., India). The dialysis bag containing 0.1 g ml^−1^ bacterial EPS was suspended in an acid-washed polyvinyl chloride (PVC) beaker containing either 4 mM [As(III)] or 10 mM [As(V)] solution with a stir bar. The PVC beaker containing the desired metal solution and the EPS-filled dialysis bag was then placed on a magnetic stirrer overnight at 28 ± 2°C. The dialysis was repeated thrice with Milli-Q H_2_O to remove any traces of weakly bound As metal ions from EPS. The metal complexed EPS solution from the dialysis bag was then transferred to clean microfuge tubes and stored at −20°C prior to AAS analysis. For blank preparation, the bacterial EPS was substituted with equal volume of Milli-Q H_2_O and incubated under similar conditions. Similarly, bacterial EPS without As treatment was used as control. For determination of *in vitro* salt sequestration by purified Exo1, flame-photometry was used. In brief, bacterial EPS was extracted from liquid 8% TSMGM and 1 g dried EPS was dissolved in 4 ml Milli-Q H_2_O. The EPS solution was then transferred to a dialysis bag of 12–14 kDa MW cut-off. The dialysis bag containing 0.25 g ml^−1^ bacterial EPS was suspended in an acid-cleaned PVC beaker containing 80 ml of 5, 15, or 20% (w/v) NaCl solution with a stir bar. The PVC beakers containing the desired salt solution and the EPS-filled dialysis bag were then placed on a magnetic stirrer overnight at 28 ± 2°C. The dialysis was repeated thrice with equal proportion of Milli-Q H_2_O to remove any traces of weakly bound Na^+^ ions from the EPS and the electrical conductivity of the H_2_O was routinely monitored after completion of every dialysis. The solution containing Na^+^-EPS complex was then dried at 60°C prior to analysis. The amount of EPS-bound Na^+^ ions were analyzed via flame-photometry (Aimil Ltd., India) as mentioned earlier. For blank preparation, the bacterial EPS was replaced with equal proportion of Milli-Q H_2_O and incubated under similar conditions whereas bacterial EPS without salt treatment was incubated in Milli-Q H_2_O and used as control. Finally, the concentration of Na^+^ ion was calculated from the standard curve generated using known concentrations of Na^+^ solution. For detection and measurement of antioxidant activity, ascorbic acid of various concentrations (1–10 mg ml^−1^) was prepared from a standard stock solution of 100 mg ml^−1^ in deionized water. Solutions of purified EPS (10 mg ml^−1^) were also prepared in deionized water. 0.05 mM of DPPH (M.W. = 394.32 g) was prepared in absolute ethanol. For preparation of standard curve, 1 ml of each ascorbic acid solution (1, 2, 4, 6, 8, 10 mg ml^−1^) was mixed with 2 ml of ethanolic DPPH (0.05 mM) and kept in dark for 30 min. Similarly, 1 ml of each EPS solution was mixed with 2 ml of ethanolic DPPH (0.05 mM) and kept in dark for 30 min. The absorbance was measured at 517 nm using deionized water as blank and DPPH. Absolute ethanol was used as reference. The % scavenging of DPPH was calculated using the following equation:

(1) $$\%\text{ Scavenging}=(A_{\text{blank}}-A_{\text{sample}}/A_{\text{blank}})\times 100$$

### Qualitative and Quantitative Estimation of Plant Growth-Promoting Metabolites Under Salt and Arsenic Stresses

Plant growth-promoting properties like phosphate solubilization, production of IAA, siderophore, ammonia, HCN, biocontrol, and nitrogen fixing abilities of the strain were investigated under normal condition and salt and As stress conditions.

#### Estimation of Inorganic Phosphate Solubilization and Organic Acid Production

The phosphate solubilization activity was determined by the protocol of Mehta and Nautiyal (2001) with modifications. Briefly, the isolate was grown in NBRIP medium (in absence and presence of NaCl and/or As) containing a pH indicator (Bromophenol Blue) for 10 days at 30 ± 2°C with continuous agitation. At 10th day, the final OD_600_ value was subtracted from the initial A_600_ value (0th day). Phosphate solubilization efficiency was determined by spot inoculating 3 μl of each isolate on Pikovskaya's (PVK) agar plate. Phosphate solubilization index (PSI) was calculated (diameter of phosphate solubilization (PS) zone on PVK agar/ growth diameter of spot inoculant) after 12 days of incubation at 30 ± 2°C. Finally, the kinetics of Ca_3_(PO_4_)_2_ solubilization mediated by each of the isolates were monitored in liquid NBRIP medium at five time-points (0, 4, 8, 12, and 16 days). Soluble Pi concentration in the medium was estimated spectrophotometrically by using the molybdenum blue method (Mukherjee et al., 2017) and the decrease in pH values over time (because of organic acid production) was also recorded.

#### Estimation of Indole-3-Acetic Acid (IAA) Production

Spectrophotometric estimation of IAA was conducted as per the method of Goswami et al. (2015). An aliquot (2 ml) of 3 to 8-day old culture supernatant grown in LB broth and 8% TSMGM supplemented with 500 mg l^−1^ of L-tryptophan (in absence or presence of As i.e., 0, 2, 4, 6 mM As) at 30 ± 2°C was transferred to a clean test tube to which 100 μl of ortho-phosphoric acid and 4 ml of Salkowski reagent (50 ml, 35% of perchloric acid (HCIO_4_), 1 ml of 0.5 M FeCl_3_ solution) were added. The mixture was incubated at room temperature (dark) for 25 min and the intensity of pink color developed was recorded at 530 nm. A standard curve of IAA (10–100 μg ml^−1^) was used for estimation of IAA produced.

#### Estimation of Siderophore Production

For the six isolates, production of siderophore was initially assessed using Chromazurol S (CAS, Loba Chemie) agar medium as described by Schwyn and Neilands (1987). Test cultures were spot inoculated (3 μl) on CAS agar plates (with or without 5% NaCl) and incubated at 30 ± 2°C. An orange halo zone around the growth confirms positive siderophore production, the diameter of which was measured after 24, 48, and 72 h. Amount of siderophore produced was further quantified using CAS-shuttle assay (Payne, 1994). Bacterial cultures were grown in King's B media in presence and absence of NaCl and [As(III)]. Samples were withdrawn and centrifuged at 10,000 rpm for 5 min. CAS assay solution was added to culture supernatant in equal proportion, mixed and allowed to stand for 20 min. Siderophore, if present, removes the iron from the dye complex, causing reduction in the intensity of blue color, which was recorded at 630 nm. For the measurements, minimal medium was used as blank and % siderophore units were calculated by the following equation:

(2) [(Ar−As)/Ar] × 100=% siderophore units

where Ar = absorbance of reference (minimal media + CAS assay solution), As = absorbance of sample.

#### Estimation of Ammonia (NH_3_) Production

Production of ammonia was determined as described in Cappuccino and Sherman (1992) with modifications. 24-h old bacterial cultures were inoculated in 10 ml peptone broth (1% NaCl) and liquid 8% TSMGM (without yeast extract) supplemented with peptone and As (2, 4, 6 mM), and incubated at 30 ± 2°C for 4–12 days with constant shaking. After incubation, 2 ml of bacterial culture was taken in an eppendorf tube and centrifuged at 10,000 rpm for 5 min. Then, 40 μl of Rochelle salt (K-Na-tartrate) and 40 μl of Nessler's reagent were added in each tube containing the supernatant. The development of yellow to dark brown color indicated the production of ammonia and absorbance was measured at 425 nm. NH_3_-N standard curve (in the range of 100–1,000 μg l^−1^) was prepared using Nesslerization spectrophotometric method to estimate the concentrations of NH_3_ produced.

#### Estimation of Hydrogen Cyanide (HCN) Production

For qualitative estimation of HCN production, Picrate assay as described by Castric (1975) was followed. The isolate was streaked on LB agar and incubated overnight. A Whatman filter paper No. 1 soaked in solution of 2% Na_2_CO_3_ and 0.5% picric acid was placed in between base and lid of the culture plate. Plate was sealed with parafilm and incubated at 30 ± 2°C for 72 h. Production of HCN was indicated by color change of filter paper from yellow to orange-brown. For quantitative estimation of HCN production, 24-h old bacterial cultures were inoculated in 10 ml LB broth and liquid 8% TSMGM (with and without As) and incubated at 30 ± 2°C for 24–48 h with constant shaking. Then, 1 ml solution containing 2% Na_2_CO_3_ and 0.5% picric acid was added to the 2-days old culture and further incubated at 30 ± 2°C with constant shaking. Then, after every 24 h, 2 ml of bacterial culture was taken in a microfuge tube and centrifuged at 10,000 rpm for 5 min. The development of yellow to brownish red color indicated the production of HCN and the absorbance was measured at 490 nm. A broth without inoculum was used as a reference. The total cyanide (in mg l^−1^) was estimated by using the following equation:

(3) $$\text{Total cyanide content }({\text{in mg l}}^{-\text{1}})\text{ = 396 }\times {\text{ A}}_{\text{490}}\text{ nm}$$

(Kumar et al., 2015).

#### Estimation of Nitrogen (N_2_) Fixation

N_2_-fixation ability was evaluated by growing isolate on N-free solid agar media without salt-supplementation like Norris Glucose N-free medium (10 g l^−1^ glucose, 1 g l^−1^ K_2_PO_4_, 0.2 g l^−1^ MgSO_4_.7H_2_O, 1 g l^−1^ CaCO_3_, 5 g l^−1^ NaCl, 0.005 g l^−1^ Na_2_MoO_4_.2H_2_O, 0.1 g l^−1^ FeSO_4_, final pH-7.0 ± 0.2) and supplemented with 1.5% (w/v) agar and in semi-solid Burk's N-free medium (BHM) adjusted to the appropriate saline concentration of 7.5% (w/v) (0.64 g l^−1^ K_2_HPO_4_, 0.16 g l^−1^ KH_2_PO_4_, 58.5 g l^−1^ NaCl, 14.73 g l^−1^ MgSO_4_.7H_2_O, 0.4 g l^−1^ CaSO_4_.2H_2_O, 8 g l^−1^ glucose, 0.001 g l^−1^ Na_2_MoO_4_.2H_2_O, 0.003 g l^−1^ FeSO_4_), and supplemented with 0.2% (w/v) agarose (Argandoña et al., 2005).

#### Determination of Biocontrol Activities

Anti-microbial activity of the isolate was tested on Potato Dextrose Agar (PDA) using agar well diffusion method (Bauer et al., 1966). Briefly, overnight culture of *Fusarium oxysporium* was inoculated into PDA by pour-plating and the 24-h old bacterial culture was placed in the well. Same experiment was repeated using pure EPS in the wells of the plates. The plates were incubated for 48 hours and observed for the zone formation.

### Germination and Growth of Salt-Tolerant Rice Seeds Treated With Whole Bacterial Cells or Purified Exopolysaccharide Under Salt and Arsenic Stresses

Seeds of moderately salt-tolerant rice variety-Jarava (IET- 15420) were obtained from Salt and Flood Resistant Paddy Research Station, Gosaba (Rice Research Station, Chinsurah, West Bengal, India). Soil for the pot assay was also obtained from paddy cultivation field of Gosaba. pH, electrical conductivity and salinity of the soil were found to be 7.2, 0.32 mS/cm and 3 PSU, respectively. Nitrogen (N), available phosphorous (P), potassium (K), total organic matter, organic carbon and total arsenic (As) contents of the soil were found to be 0.10%, 14.20 mg kg^−1^, 2267.28 mg kg^−1^, 1.12%, 0.85%, and 2.96 mg kg^−1^, respectively. Rice seeds were surface sterilized by immersion into solution A (70% ethanol and 0.3% Tween 80) for 5 min, and then in solution B (3% sodium hypochlorite and 0.3% Tween 80) for 15 min and then washed thrice with sterile distilled water. For germination, rice seeds were soaked in sterile distilled water for 12 h, and then covered with a clean wet cloth for 12 h. *Halomonas* sp. Exo1 was cultivated (either alone or in consortia with other 5 *Halomonas* strains) in LB broth for 24–48 h and centrifuged. The collected bacterial cells were washed three times with deionized water and re-suspended in deionized water to obtain ~10^8^ CFU/ml (A_600_ nm = 0.5). Each petri plate containing one sheet of paper was moistened with 10 ml of deionized water. For bacterization, 2 ml of test bacterial suspension was initially applied to the rice seeds in each treatment plates and 2 ml of deionized water was added to the control plate. Germination was carried out for 15 days under artificial light for 12-h photoperiod with a light intensity of 2,100 Lux, a temperature of 28 ± 4°C, and humidity of 54 ± 8% and percentage of germination and growth parameters of germinated seedlings were measured. Post-germination plantlets were directly sown in watertight brown plastic pots (16 cm diameter × 9 cm height). The *in vivo* pot trials were carried out in triplicates under natural light with a 15-h photoperiod during the month of Nov to Feb when the average temperature and humidity were 28 ± 4°C and 50 ± 10%, respectively. Pots were filled with 1,000 g of saline treatment soil containing 1 g NaCl (EC at 2–4 dS m^−1^), urea (90 kg N ha^−1^), muriate of potash (11 kg K_2_O ha^−1^) and single superphosphate (40 kg P_2_O_5_ ha^−1^). In addition, 20 mg of NaAsO_2_ and 2 g of NaCl were added per 1,000 g of saline treatment soil in case of As and salt treatment pots, respectively. In each pot, 6 surface-sterilized 15-days old seedlings were uniformly transplanted at the depth of ~2.5 cm below the soil surface and each seedling was inoculated with 5 ml test bacterial suspension or 0.2 g of pure Exo1. For negative control (plants germinated from non-coated seeds), soil was not treated with the test bacteria or bacterial EPS. Rice seedlings were harvested (uprooted) after 15 days of plantation/sowing, and vegetative parameters (root and shoot lengths, fresh and dry weights) were analyzed. Oven-dried soil and plant samples (roots and shoots) were grounded to powder, acid digested, and [As(III)] contents were analyzed by AAS (NovAA 350, Germany). Bioaccumulation Factor and Translocation Factor of [As(III)] and germination index were calculated by the following equations:

(4) $$\text{Bioaccumulation Factor }(\text{BAF})\;={\text{ C}}_{\text{root}[\text{As}(\text{III})]}{\text{/C}}_{\text{soil}[\text{As}(\text{III})]}$$

(5) $$\text{Translocation Factor }(\text{TF})\;={\text{ C}}_{\text{shoot}[\text{As}(\text{III})]}{\text{/C}}_{\text{root}[\text{As}(\text{III})]}$$

(6) $$\begin{array}{l} \text{Germination Index }(\text{GI})\;=\;(\text{Number of seeds germinated/}\\ \;\text{total number of seeds})\;\times \text{ 100} \end{array}$$

Total Kjeldahl Nitrogen (TKN) and phosphate (${\text{PO}}_{4}{}^{3-}$) contents in acid-digested roots, shoots and pot soil from each treatment conditions were analyzed using methods described in APHA (2012).

### Statistical Analysis

Statistical significance was analyzed with respect to control in all the cases and performed using One-Way ANOVA. “a,” “b,” and “c” mean that the result is significant at *p* < 0.01, *p* < 0.05, and *p* < 0.1, respectively, and *p* > 0.1 has been considered less or insignificant and is denoted as “d” in the figures. All statistical analyses were done using SPSS 12.0 for Windows (SPSS Inc., USA). Plant growth-promoting activities were performed in a completely randomized block design. Each experiment was repeated three times. The differences among the mean values were determined using Duncan's Multiple Range Tests (DMRTs) at *P* < 0.05. The results have been graphically presented using GraphPad Prism Software 7 for Mac OS (San Diego, California, USA) while Statistic Analysis System (SAS 9.1) was used for DMRT analysis.

### Nucleotide Sequence Accession Numbers

The accession numbers of the 16S gene sequences of the isolates *Halomonas* sp. Exo1, *Halomonas* sp. Exo2, *Halomonas* sp. Exo6, *Halomonas* sp. Exo7, *Halomonas* sp. Exo8, and *Halomonas* sp. Exo9 obtained from GenBank were KT238978.1, KT238979.1, KT238983.1, KT238984.1, KX026971.1, and KX026972.1 respectively.

## Results

### Physico-Chemical Analysis of *Avicennia marina* Rhizosphere Soil

The rhizosphere soil was found to contain 1.427% of NaCl and 1.96 ppm of As. The detailed physico-chemical properties of the soil sample (pH 7.0) are listed in the Supplementary Table S1.

### Isolation and Identification of Exopolysaccharide Producing Halo-Rhizobacteria

**Figure 1:**
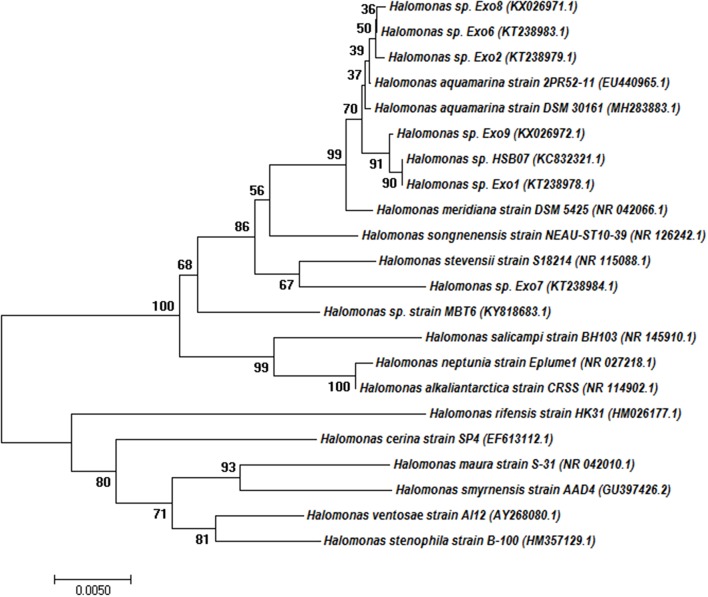
Phylogenetic tree based on 16S rRNA gene sequences obtained by the neighbor-joining (NJ) method showing the phylogenetic relationship of the *Halomonas* sp. Exo1 with the related species. The percentage of replicate trees in which the associated taxa clustered together in the bootstrap test (1,000 replicates) is shown next to the branches. Branches corresponding to partitions reproduced in <50% bootstrap replicates are collapsed. Accession numbers are written after the species or strain names.

Out of the 14 plant growth-promoting halo-rhizobacterial strains growing in 4 mM [As(III)] supplemented 8% TSMGM, six strains were initially selected due to their production of EPS. 16S rDNA sequence analysis showed all the six strains belong to the members of the genus *Halomonas*. Figure 1 shows the evolutionary relationship of the *Halomonas* isolates among themselves and with other EPS producing or non-EPS producing *Halomonas* strains.

Phylogenetic analysis demonstrated that *Halomonas* sp. Exo1 is more closely related to *Halomonas* sp. Exo9 than *Halomonas* sp. Exo2, *Halomonas* sp. Exo6, *Halomonas* sp. Exo8 and *Halomonas* sp. Exo7. *Halomonas* sp. strain Exo1 showed 100% identity to non-EPS producing *Halomonas* sp. HSB07 (KC832321.1) (Liu et al., 2013) but only 94% similarity with EPS producing strain *Halomonas ventosae* Al12 (Martínez-Cánovas et al., 2004) and 93% identity to three strains: mauran (EPS) producing *Halomonas maura* strain S-31 (NR_042010.1) (Bouchotroch et al., 2001), *Halomonas rifensis* strain HK31(Amjres et al., 2011) and *Halomonas smyrnensis* strain AAD4 (Poli et al., 2013).

**Figure 2:**
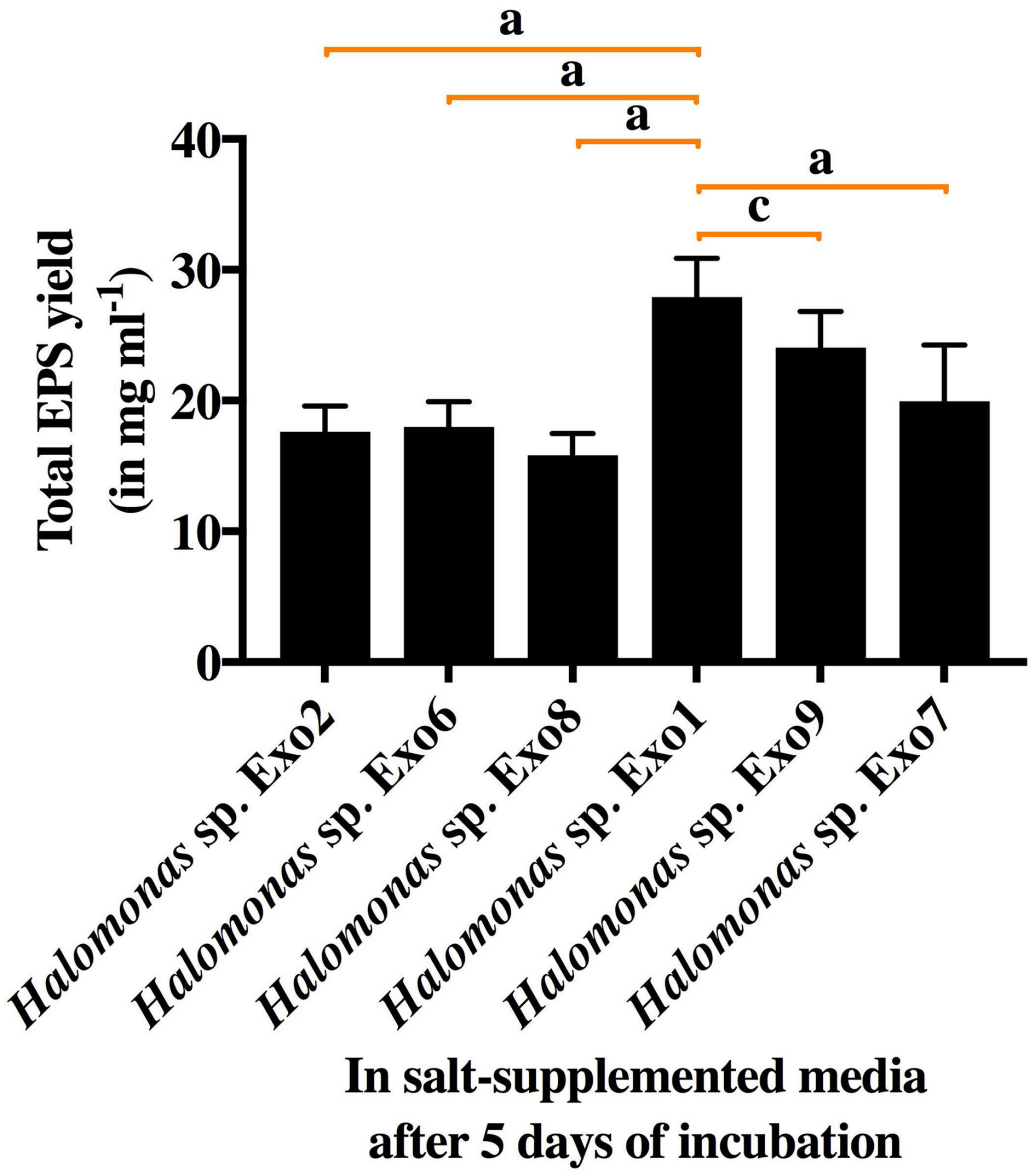
Total EPS yield from 6 selected *Halomonas* strains in salt-supplemented medium (8% TSMGM) after 5 days of incubation. Vertical bars represent mean ± standard deviation of triplicate measurements. Statistical significance of EPS yield from *Halomonas* sp. Exo1 was measured separately with each of the 5 isolates and shown accordingly.

All the 6 *Halomonas* strains produced water-soluble EPS of varying amounts (15.8–27.9 mg ml^−1^) in 8% TSMGM (Figure 2). Among them, *Halomonas* sp. strain Exo1 was selected for further study based on its highest yield of EPS production.

**Figure 3:**
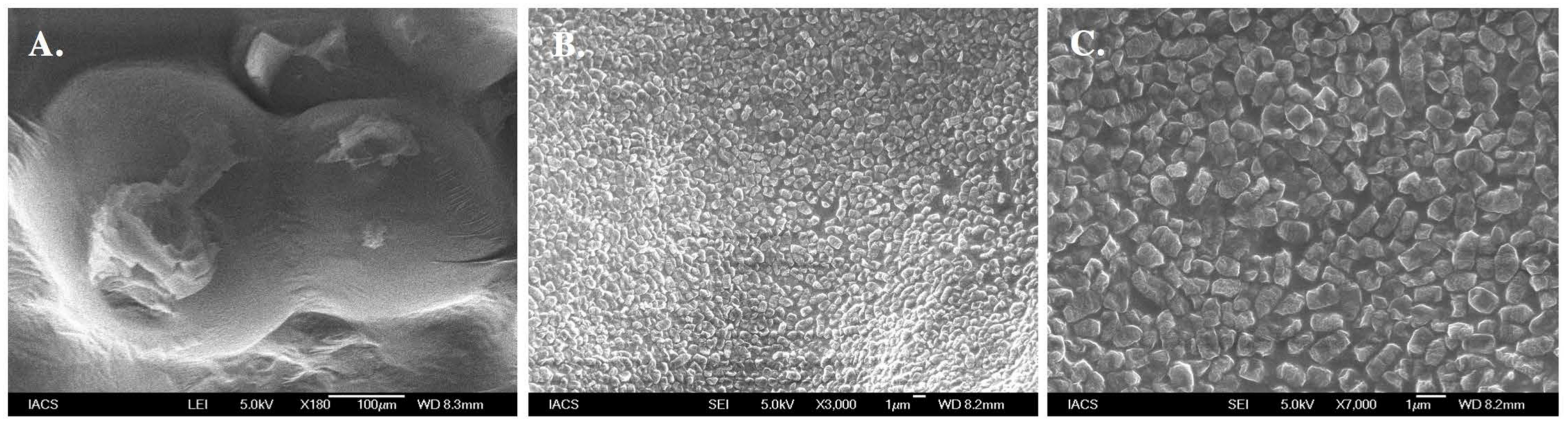
Morphological analysis of EPS producing *Halomonas* sp. Exo1. Field Emission Scanning Electron Micrograph (FESEM) images of *Halomonas* sp. Exo1 displaying loosely attached polymeric substances on its cell surface **(A)** with zoomed in images of numerous crystal like structures of cell-bound EPS on the bacterial cell surface **(B,C)**.

Figure 3 shows Field Emission Scanning Electron Microscopy (FESEM) images of the topography of EPS coated cell of *Halomonas* sp. Exo1 in which a single short rod-shaped cell with polymer-like substances forming a thin matrix over the cell surface was seen (Figure 3A). In addition, closer microscopic observation revealed the presence of numerous irregular crystal-like structures probably of cell-bound EPSs on the cell surface (Figures 3B,C). Instead of SEM, FESEM was selected as this technique provides a clearer, less electro statically distorted image with nanometer level spatial resolution that is three to six times better than SEM and allows measurements of fine structural and micromechanical properties of sample. Further it minimizes sample charge up and damage as it is operated at lower electron-accelerating voltages, and gives better images of immediate biological surfaces due to a reduction in electron penetration.

### Characterization of the Plant Growth-Promoting Rhizomicrobes Toward Abiotic Stress Tolerance: Salt and Arsenic

**Figure 4:**
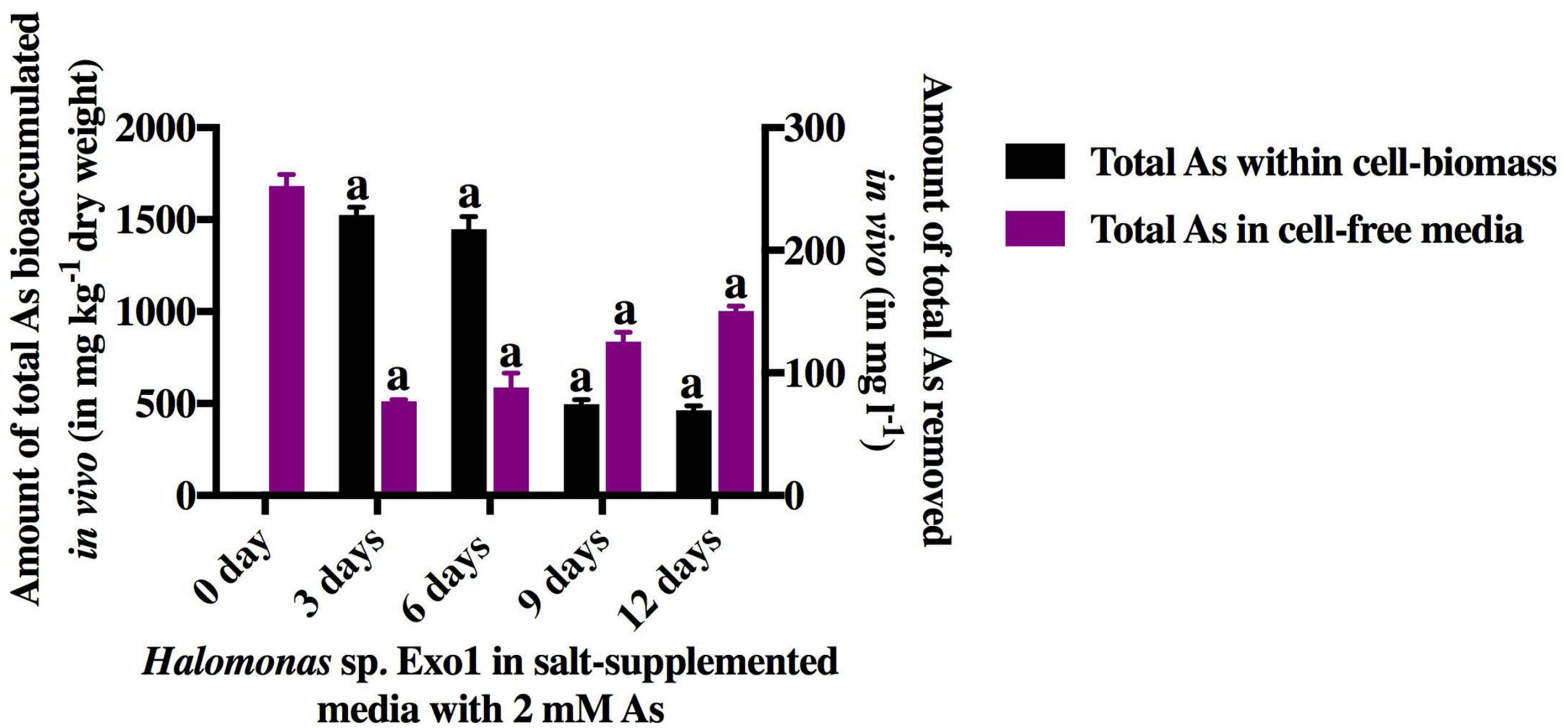
*In vivo* time-dependent arsenic bioaccumulation by live cells. The amount of total [As(III)] in ambient media and total [As(III)] bioaccumulated within cell-biomass of *Halomonas* sp. Exo1 are represented as mg l^−1^ and mg kg^−1^ (dry weight), respectively. Vertical bars represent mean ± standard deviation of triplicate measurements values. 252.42 mg l^−1^ As was detected in 0-day cell-free 8% TSMGM containing 2 mM [As(III)] (positive control for As in ambient media). 0.83 mg kg^−1^ (dry weight) trace As was detected in 3-day cell-pellet grown in 8% TSMGM without As (negative control for As bioaccumulation).

The isolated PGPR, *Halomonas* sp. Exo1 was found to be euryhaline facultative mesohalophilic in nature and could tolerate wide range of salt concentrations (upto 20% NaCl and 24% total salt). The strain was found to accumulate fairly large amounts of osmolytes when grown in 8% TSMGM. The order of accumulation of osmolytes in 8% TSMGM grown cells of *Halomonas* sp. Exo1 were in the following order: Na^+^ (42,839 mg kg^−1^ dry weight) > Mg^2+^ (29,288.1 mg kg^−1^ dry weight) > K^+^ (13,601.7 mg kg^−1^ dry weight) > Ca^2+^ (1,847.46 mg kg^−1^ dry weight). On the other hand, in the LB broth grown control cells, Na^+^ accumulation was 6,310 mg kg^−1^ dry weight while that of K^+^, Mg^2+^, and Ca^2+^ were found to be below detectable limits (Supplementary Figure S1). In the 8% TSMGM medium, amount of Na, Mg, K, and Ca cations were 6.4, 1.733, 0.187, and 0.013% respectively. Although presence of organic osmolytes or exact mechanism of osmoregulation in *Halomonas* sp. Exo1 was not investigated but higher intracellular concentration of Na^+^ than K^+^ ions indicates that it is following the same haloadaptation mode as most other moderate halophiles displaying “low salt in” strategy (Oren, 2008). Although the strain could tolerate up to 8 mM [As(III)], increasing concentration of As lowered growth rate and salt tolerance. Supplementary Figure S2 shows growth profiles of *Halomonas* sp. Exo1 in presence of the abiotic stresses and MIC values toward salt and As tolerances. The As tolerance ability of *Halomonas* sp. Exo1 is believed to be partly due to detoxification ability to oxidize more toxic form of [As(III)] to less toxic form of [As(V)] as observed biochemically (Supplementary Figure S3) and partly due to bioaccumulative ability of [As(III)] as evidenced by AAS study of the acid digested cell biomass grown in As supplemented medium (Figure 4). In *Halomonas* sp. Exo1, maximum amount of As bioaccumulation occurred during early to late log phase of growth where 252.4 mg l^−1^ As was detected in the cell-free medium on day 0 in which 2 mM [As(III)] was originally added. Highest accumulation of As (1527 mg kg^−1^ dry weight) was found in the pellet on day 3 after which it started to decline. Arsenic level in the media started to increase and reached a steady state during late stationary to death phase. Bacterial culture grown in 8% TSMGM for three generations in the absence of As showed only trace amount of As in the 3-days old cell-pellet (0.83 mg kg^−1^ dry weight) and served as negative control. The whole experiment was conducted in the presence of salt which was found to enhance HM tolerance. It has been hypothesized that the decrease in As level within cell-pellets could be owing to cell autolysis during death phase resulting in the release of As back into the growth medium.

To further proof biomass mediated physical adsorption of As, in addition to live cells, dead bacterial biomass mediated [As(III)] biosorption was quantified. The dead cell-biomass of *Halomonas* sp. Exo1 induced 20.91% depletion of the initial 4 mM sodium arsenite and the As content of the cell-free extract post incubation was 45.38% (considering the initial metal concentration of the metal solution to be 100% prior to incubation with dead bacterial cell). The loosely bound As (adhering to the dead cell surface) must have been removed during the washing steps. The mass of [As(III)] bioadsorped was 43 mg kg^−1^ (dry weight) of dead cell-biomass. The percentages of As in the supernatants and the bacterial pellets may not have reached 100%. Some As may be lost during multiple washing of the cells with deionized water. The washing may have caused release of some cell-bound EPS (with bound As) into the washed-out fraction. This washed-out fraction would not be present in the supernatant, as the supernatant was filtered prior to quantification in order to suppress the potential contribution of the secreted bacterial EPS to As adsorption. In addition to As, stress responses of *Halomonas* sp. Exo1 toward 10 other heavy metals in LB and salt media were also studied. The MIC values of the strain in 8% TSMGM were 8 mM for [As(III)], 140 mM for [As(V)], 15 mM for [Cr(III)], 2 mM for [Cr(VI)], 1 mM for [Co(II)], 0.4 mM for [Pb(II)], 1 mM for [Cu(II)], 0.4 mM for [Zn], 500 mM for [Mo], 90 mM for [Mn], 2.0 mM for [Ni(II)], and 0.5 mM for [Cd]. In the absence of salt (i.e., in LB media), the MIC values were 1 mM for [As(III)], 150 mM for [As(V)], 8 mM for [Cr(III)], 1 mM for [Cr(VI)], 1 mM for [Co(II)], 0.5 mM for [Pb(II)], 1.5 mM for [Cu(II)], 2 mM for [Zn], 550 mM for [Mo], 20 mM for [Mn], 2.5 mM for [Ni(II)], and 0.2 mM for [Cd]. So, for certain metals like [As(III)], Cr, Cd, and Mn, MIC values dropped in the absence of salt. This indicated the role of salt in increasing resistivity toward these metals in *Halomonas* sp. strain Exo1. In later studies, it was found that EPS secreted by the strain in presence of salt was responsible for their increased tolerance toward selected HMs. This result is significant as this is the first report, which shows that salt can play a role in increasing metal tolerance of selected HMs.

### Exopolysaccharide Production Under Salt and Arsenic Stresses

Effects of increasing concentration of salt and As on EPS yield in *Halomonas* sp. strain Exo1 is shown in Supplementary Figure S4. It may be mentioned here that in the absence of salt or with 0.5% NaCl supplementation in LB broth, detectable EPS production was not noticed in *Halomonas* sp. Exo1. Interestingly, EPS production was detected when LB broth was amended with 1% NaCl. EPS production increased with increase in NaCl concentration up to 20%, where maximum EPS yield was observed. A sudden sharp fall in EPS production was noticed when NaCl concentration was increased to 22.5%. It is believed that a breakdown of osmo-tolerance caused growth inhibition and decrease in EPS production. Effect of As stress on the production of EPS (both cell-free and cell-bound) in 8% TSMGM was depicted in *Halomonas* sp. Exo1 (Supplementary Figure S4B). It was found that EPS yield increased with increasing [As(III)] concentration up to 6 mM. However, 8 mM [As(III)] caused significant decrease in EPS yield. Amount of cell-bound EPS was negligible in comparison to cell-free EPS both in absence and presence of different concentrations of [As(III)], suggesting that majority of the EPS produced in *Halomonas* sp. Exo1 was secreted in the surrounding medium. Time-dependent study in 8% TSMGM containing 2 mM As showed that total EPS yield increased with days elapsed and maximum yield was observed during late stationary phase of bacterial growth.

### Biophysical Characterization of the Exopolysaccharide

**Figure 5:**
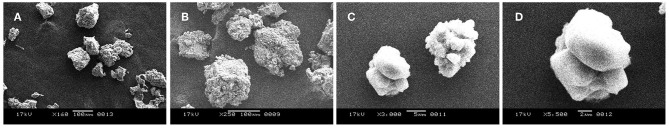
Scanning Electron Micrograph (SEM) images of purified Exo1 showing compact globular nature **(A–D)**.

The secreted EPS was found to be soluble in water. The salinity and electrical conductivity of the crude EPS prepared in deionized H_2_O (20 mg ml^−1^) prior to desalting was 17 PSU and 25 ± 0.2 mS/cm, respectively. With an increase in As stress (6 mM) under saline condition, the salinity and electrical conductivity of crude Exo1 became18 PSU and 26.4 ± 0.5mS/cm. After desalting, the extracted EPS showed salinity and electrical conductivity of 0.5 PSU and 0.083 mS/cm, respectively. This observation suggests that the cell-free EPS is capable of quenching mixed sea salts from the cultivating medium. Total neutral-carbohydrate in cell-free and cell-bound EPS were 0.58 and 20.87 μg μl^−1^, respectively. Concentrations of proteins in cell-free and cell-bound EPS were 76.12 and 75.53 μg μl^−1^, respectively. Lipid estimation showed 25 mg g^−1^ lipids content in total EPS while that of sulfate content was found to be 90.36 mg g^−1^. Scanning electron micrograph revealed that the purified EPS was compact and globular in nature (Figure 5), which is a characteristic of plasticized films (Wang et al., 2010).

**Figure 6:**
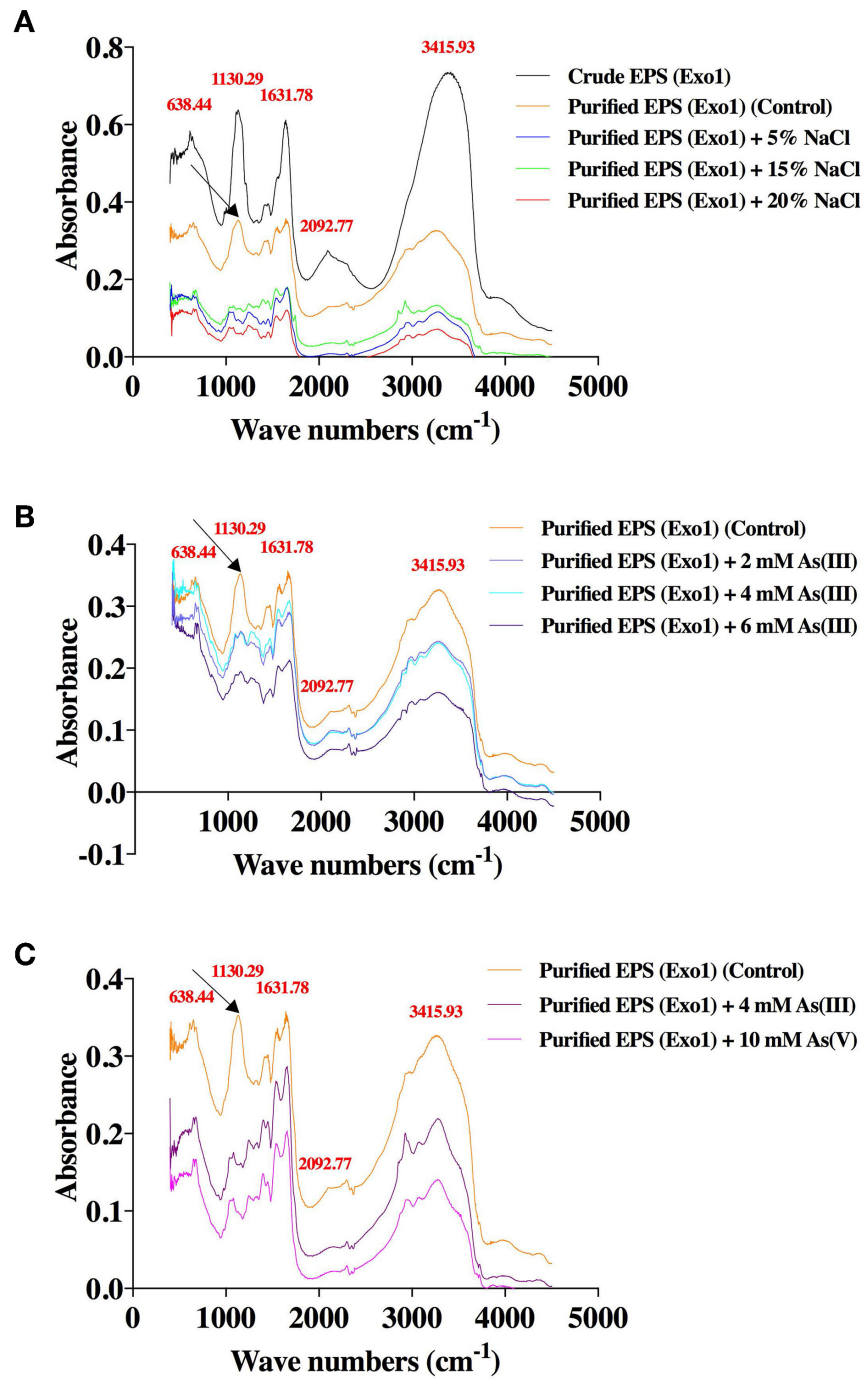
Effects of salt and arsenic stresses on the FT-IR spectra of functional groups of purified Exo1 done in solid (KBr) phase. A. *In vitro* assay: Exo1 extracted from cells grown in 8% TSMGM media was incubated with 5, 15, and 20% NaCl. B. *In vivo* assay: Purified Exo1 was extracted from bacteria grown in 8% TSMGM containing different concentrations of [As(III)]. C. *In vitro* assay: Purified Exo1 was extracted from bacteria grown in salt-supplemented (8% TSMGM) media without As and purified EPS was incubated *in vitro* with 4 mM [As(III)] and 10 mM [As(V)]. The wave numbers of distinct peaks in cm^−1^ are shown in red with respect to crude/purified Exo1. An arrow demarcates a peak that is absent in salt-treated purified Exo1 **(A)** and in As-treated purified Exo1 in case of both *in vivo* **(B)** and *in vitro* **(C)** assays.

The XRD profile of EPS exhibited non-crystalline nature of the substance with major characteristic diffraction peaks at 32.1 and 45.6 Å containing inter planar spacing (d-spacing) of 2.786 and 1.988 Å respectively (Supplementary Figure S5). To understand the role of EPS in salt and As sequestration, pure untreated EPS and pure EPS incubated with NaCl and As were analyzed by FT-IR (Figure 6). The adsorption of heavy metals or inorganic osmolytes by EPS is energy independent, and could occur through interaction between metal cations and functional groups of the EPS. So, the effects of salt and As stresses the functional groups of the purified EPS were determined. The FT-IR analysis of untreated pure EPS showed a broad stretching peak at 3415.93 cm^−1^ (range 3,600–3,200 cm^−1^), corresponding to hydroxyl group(s) (Kavita et al., 2013; Wang et al., 2015), which is generally the characteristic of a carbohydrate ring (Lim et al., 2005; Kumar et al., 2011) while a small peak at 2092.77 cm^−1^ corresponds to the presence of free carboxyl groups (Osman et al., 2012). Presence of an asymmetric medium stretching peak at 1,631 cm^−1^ may correspond to the ring stretching of mannose or galactose (Freitas et al., 2009) or to the stretch vibration of C = O bond (François et al., 2012; Zhang et al., 2017). The absorption peaks ranging from 1,000 to 1,200cm^−1^ were designated to C-O-C and C-O (Freitas et al., 2009; Kavita et al., 2011). A peak at 1130.29 cm^−1^ (1,000–1,125 cm^−1^) may be attributed to O-acetyl ester linkage bond of uronic acid (Bramhachari and Dubey, 2006). The absorption peak at 638 cm^−1^ (690–515 cm^−1^) corresponds to stretching of alkyl-halides (Kavita et al., 2011). Interestingly, both salt and metal laden EPS from *in vivo* and *in vitro* studies clearly reveal the absence of the peak at 1130.29 cm^−1^ (Figures 6A–C). This shows significant association of acetyl group of EPS in Na^+^ and As binding as has been postulated by another report where CO group was involved in divalent cation binding (Ozturk et al., 2014).

Analysis of the composition of crude EPS, purified EPS and purified EPS treated with different concentrations of salt produced by *Halomonas* sp. Exo1 by SEM-EDX revealed the presence of common elements (C, N, O) and osmolytes (Na, K, Mg, Ca), apart from chlorine, sulfur and phosphorous residues. Supplementary Table S2 shows weight and atomic percentages of these elements. Accumulation of these osmolytes within EPS perhaps occurred during EPS production in salt-supplemented media, which contained several sea salts including NaCl, KCl, MgCl_2_, MgSO_4_, and CaCl_2_. However, the weight % and atomic % of different elements between crude and purified Exo1 showed marked variations, probably owing to the removal of considerable amounts of salts and impurities like trace metals from crude EPS during desalting and purification processes. *In vitro* assays of purified EPS laden with NaCl using salt solutions with different NaCl concentrations showed an increase of Na % weight from 1.13 of control to 2.95 for 5% NaCl and to 3.19 for 15 % NaCl concentration. For atomic weight, the values were 0.67 (control), 1.78 (5% NaCl), and 1.97 (15% NaCl).

### Functional Characterization of the Exopolysaccharide

**Figure 7:**
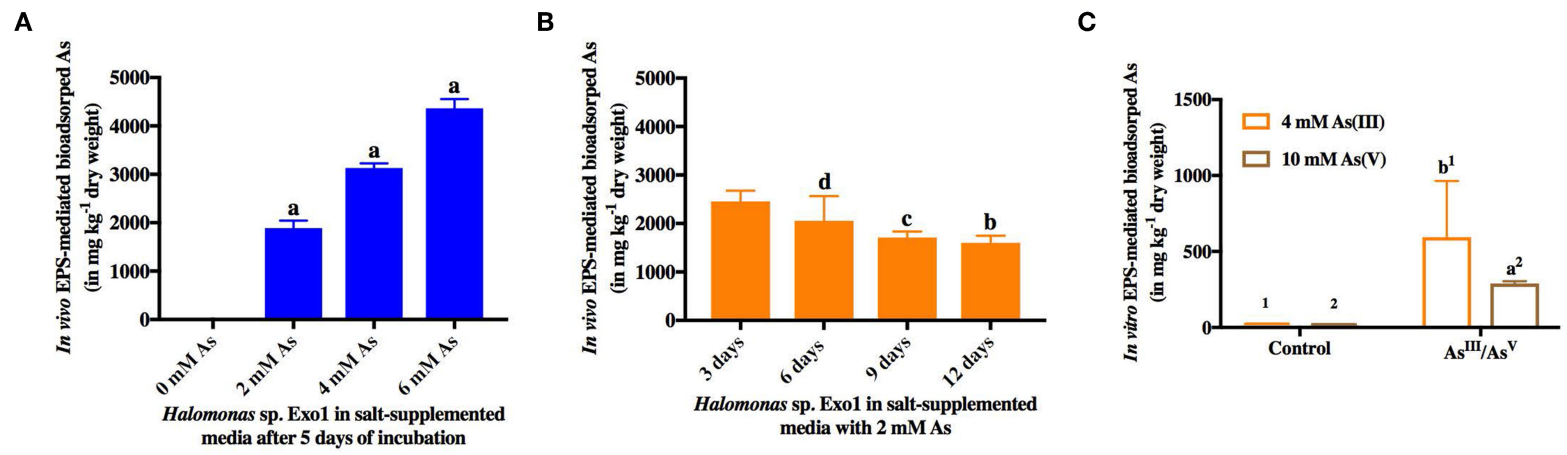
*In vivo* and *in vitro* EPS-mediated bioadsorption of arsenic. *In vivo* dose-dependent (0, 2, 4, 6 mM) **(A)** and time-dependent (3, 6, 9, 12 days) **(B)** arsenic sequestration from ambient salt media by EPS from *Halomonas* sp. Exo1. 0.86 mg kg^−1^ (dry weight) [As(III)] was detected in 5-day EPS from 8% TSMGM without As while 2.46 mg kg^−1^ (dry weight) trace [As(III)] was detected in 3-day EPS from 8% TSMGM without As (negative control) **(C)** *In vitro* bioadsorption of arsenic by Exo1. The amounts of bioadsorped [As(III)] and [As(V)] in mg kg^−1^ dry weight of purified EPS incubated overnight at 4°C in solutions containing 4 mM [As(III)] and 10 mM [As(V)], respectively. Exo1 without [As(III)]/[As(V)] treatment was used as control. Error bars represent mean ± standard deviation of triplicate values.

Finally the role of EPS in salt and As sequestration was confirmed through *in vivo* and *in vitro* arsenic and salt bioadsorption. In the *in vivo* As bioadsorption assay, purified EPS was found to be capable of sequestering [As(III)] in a dose and time-dependent manner (Figure 7). Arsenic dose-dependent study showed that the amount of EPS-mediated bioadsorped [As(III)] increased ~two-folds with every two-fold increase in [As(III)] concentration (0, 2, 4, 6 mM) and as such showed a linear correlation (Figure 7A). It has been observed that 5-day EPS from 8% TSMGM without As (negative control) contained only 0.86 mg kg^−1^ (dry weight) [As(III)]. Moreover, the results from time-dependent study showed a decrease in [As(III)] bioadsorption with increase in time, and after a period of 9 to 12 days it reached a level of saturation. The amount of [As(III)] bioadsorped by bacterial EPS from its ambient medium with time is shown in Figure 7B. A trace amount [2.46 mg kg^−1^ (dry weight)] of [As(III)] was detected in 3-day EPS from 8% TSMGM without As (negative control). In the *in vitro* EPS-mediated As bioadsorption assay, purified EPS was found to be capable of sequestering both the species of As [As(III) and As(V)]. On the other hand, only trace amounts of [As(III)] (2.86 mg kg^−1^ dry weight) and [As(V)] (1.46 mg kg^−1^dry weight) were detected in controls. Although, sequestration of [As(III)] from 4 mM NaAsO_2_ solution was higher (593.81 mg kg^−1^ dry weight) than [As(V)] sequestered from 10 mM Na_3_AsO_4_ solution (289.07 mg kg^−1^ dry weight), however, the values were not statistically significant (Figure 7C).

**Figure 8:**
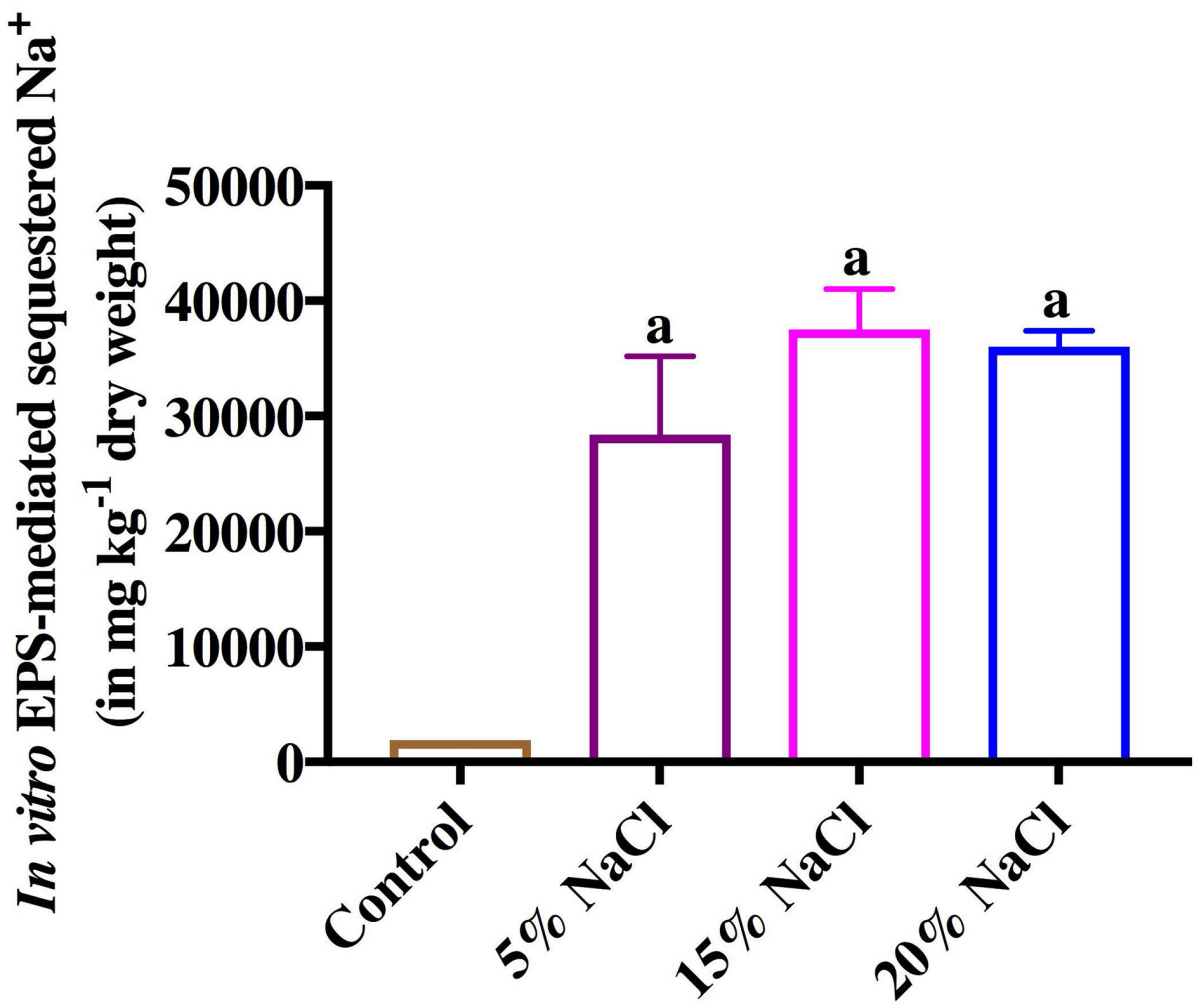
*In vitro* Exo1 mediated Na^+^ sequestration. Bars show the amounts of bioadsorped Na^+^ in mg kg^−1^ dry weight of purified EPS, which was incubated overnight at 4°C in solutions containing 5, 15 and 20% NaCl (w/v). EPS without salt treatment (0% NaCl) was used as control. Error bars represent mean ± standard deviation of triplicate values.

Like As, *in vitro* EPS-mediated salt (Na^+^) sequestration assay showed that the purified EPS from *Halomonas* sp. Exo1 can sequester Na^+^ ions from salt solutions with different NaCl concentrations (5, 15, and 20%). The amount of EPS-bound Na^+^ was found to increase with increase in NaCl concentration in the solution (28,390 mg kg^−1^ dry weight for 5% NaCl and 37,500 mg kg^−1^ dry weight for 15% NaCl), and reached a saturation level (36,000 mg kg^−1^ dry weight) at 20% NaCl. Although, the control (EPS without salt treatment) showed some amount of ionic Na^+^ (1,910 mg kg^−1^ dry weight) probably owing to residual indigenous Na^+^ that remained bound with the EPS molecules during EPS production in salt media, however, this amount was significantly less compared to the EPS-bound Na^+^ obtained under the salt-treated conditions (Figure 8).

Antioxidant ability of the pure EPS was also calculated and the percentage DPPH-free radical scavenging activity for cell-free and cell-bound EPS was found to be 33.75 and 34.03%, respectively (Supplementary Figure S6).

### Estimation of Essential Plant Growth-Promoting Properties Under Salt and Arsenic Stresses

*Halomonas* sp. Exo1 was positive for phosphate solubilization (phosphate solubilization index 2), IAA, siderophore, NH_3_ and HCN production and showed biocontrol activity against one phytopathogenic fungus *Fusarium oxysporium*. Although the isolate showed N_2_-fixing abilities as determined by growth in N-deficient media, however, the presence of *nifH* genes could not be established by PCR-based method. All plant growth-promoting properties except N_2_ fixation and biocontrol activity were analyzed under two types of abiotic stresses: salt and As. Supplementary Figure S7 shows images of the results obtained from biochemical assays on various PGP traits. Supplementary Figure S8 shows the effect of salt and As on PGP properties of the strain. OD shift assay in salt-free and salt-supplemented NBRIP-BPB media (NBRIP with bromophenol blue) showed that salt had positive impact on phosphate solubilization but As exhibited negative impact (Supplmentary Figure S9). Strain Exo1 showed maximum Pi solubilization of 286.44 μg ml^−1^ and the final media pH dropped to pH 5.36 after 8 days of growth indicating organic acid production. Similar to Pi solubilization, IAA production was more in salt-supplemented media (8% TSMGM) than in salt-deficient media. However, in salt-supplemented media under [As(III)] stress (2, 4, and 6 mM), production of IAA decreased with increase in As concentration suggesting that As stress was inhibitory to IAA production. Ammonia and HCN production were decreased under both salt and As stress conditions. So, IAA, ammonia and HCN production can be ruled out to promote enhanced plant growth under combined pressure of salt and arsenic. In salt-supplemented media, although siderophore production was initially less compared to salt-deficient media but gradually increased over time and finally became comparable after 72 h of growth. Interestingly, siderophore production was enhanced in presence of [As(III)] stress (2, 4 and 6 mM) in salt-supplemented media.

### Effects of Salt and Arsenic on Rice Growth Promotion by *Halomonas* Strains and Their Secreted Exopolysaccharide

**Figure 9:**
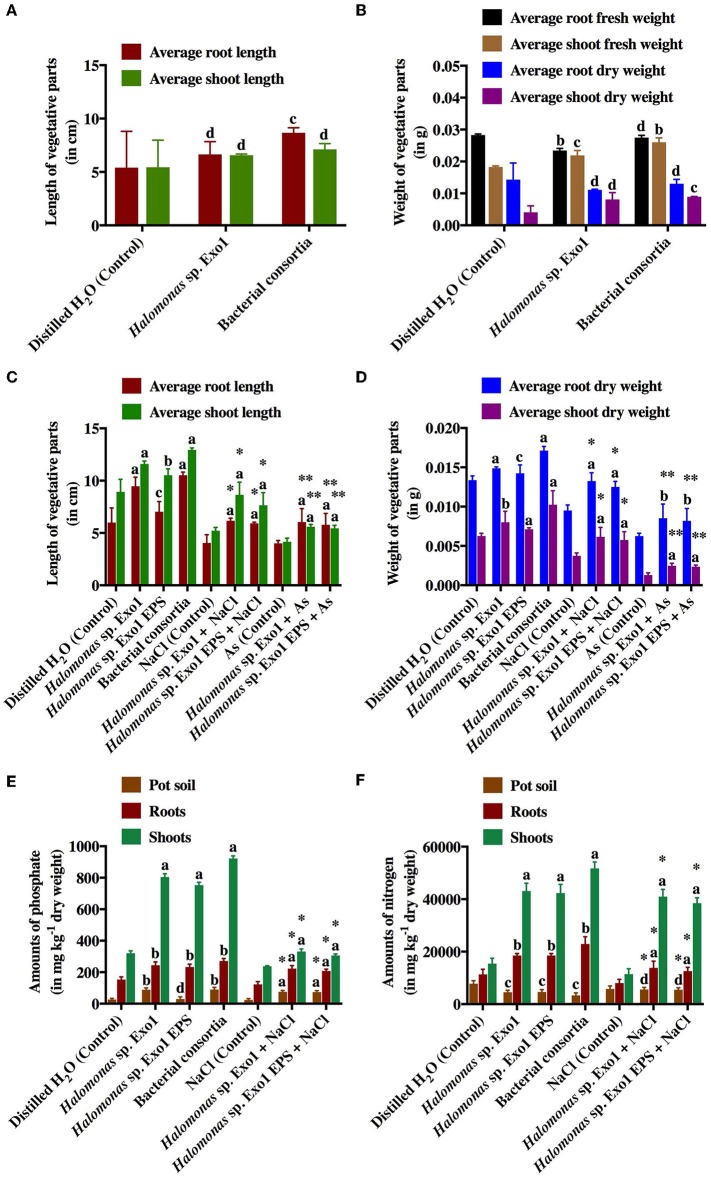
Effects of selected halo-rhizobacteria and purified Exo1 on the germination, growth and nutrient uptake ability of salt-tolerant rice under salt and arsenic stresses. Average root and shoot lengths **(A)** and average fresh and dry weights of roots and shoots **(B)** of germinated rice seeds in plate-based germination assay. Average root and shoot lengths **(C)** and average dry weights of roots and shoots **(D)** of 15-day old germinated rice seedlings in pot-based growth assay. Amounts of phosphate (${\text{PO}}_{4}{}^{3-}$) **(E)** and nitrogen (N_2_) **(F)** uptaken by the roots and shoots of 15-day old germinated rice seedlings in pot-based growth assay. Error bars represent mean ± standard deviation of triplicate values. * denotes statistical significance with respect to NaCl (control) whereas ** denotes statistical significance with respect to As (control).

Laboratory-scale plate and pot-based bioassays were conducted for germination and growth of moderately salt-tolerant rice variety (Jarava) with *Halomonas* sp. Exo1 and purified EPS. The salt-tolerant rice seeds showed good germination in distilled water (control). Seed germination stopped completely in the presence of NaCl (data not shown). As freshwater germination is must for Jarava variety, no germination was observed when seeds were incubated with bacteria grown in presence of salt. After 15 days of bacterization, the isolate either alone or in consortia showed little effect on germination percentage. In presence of Exo1 and bacterial consortia, the germination indices were calculated to be 83 and 82%, respectively whereas the control plates containing only water-germinated seedlings showed 80% germination. Similar results were observed when average lengths and average (fresh and dry) weights of roots and shoots of germinated seedlings (bacteria-treated) were measured and compared with the untreated control (Figures 9A,B). This is perhaps due to the fact that in presence of the microbes, the germination of few rice seeds was enhanced while others either remained unaltered or exhibited poor germination. Addition of 2 mM [As(III)] caused 0% germination in the control and 20% in the test plates. This slight increase in germination in the presence of As in the Exo1 treated test plates may be attributed to the microbes. The growth performances of the plants in pots (root and shoot lengths, fresh and dry weights of roots and shoots) were highest in the consortia treatment followed by *Halomonas* sp. Exo1 and lowest in untreated control. However, increase in NaCl stress (twice with respect to control) in bacteria-treated pots resulted in slight decrease in the overall growth performances, which were observed in normal unstressed plants (Figures 9C,D). Uninoculated plants showed no extra growth under this NaCl stress. Interestingly, when pot soil was amended with EPS instead of microbe, growth performance parameters of the plantlets were significantly increased as compared to the H_2_O-treated control soil. Furthermore, N_2_ and ${\text{PO}}_{4}{}^{3-}$ contents in the roots and shoots were found to be higher in treated plants (bacteria-inoculated plants and plants grown in pots containing EPS) than in control (Figures 9E,F). The amounts of N_2_ and ${\text{PO}}_{4}{}^{3-}$ in the pot soil for all treatment conditions were also comparable. Increased ${\text{PO}}_{4}{}^{3-}$ content is probably the result of the Pi solubilization ability of the PGPR isolates that may have released soluble P from the native inorganic phosphate present in the pot soil. However, the cause of increased N_2_ uptake is not clear. This may be either due to direct N_2_-fixation ability of the strains, which could not be fully confirmed in this study or N_2_ uptake-promoting ability of the phosphate solubilizing bacteria (PSBs). Supplementary Figure S10 shows images of rice growth promotion assay. Again, it was observed that all control plants that germinated normally died within 24 h in the pots amended with 20 mg kg^−1^ [As(III)]. But bacteria-treated and/or EPS-treated plants survived for 5 more days with poor growth performances, suggesting prominent metal toxicity (Figures 9C,D). Additionally, in order to confirm As toxicity the roots and shoots were separately processed for estimation of [As(III)] concentrations. Maximum accumulation of As was noticed in shoots (15 ± 0.42 mg kg^−1^) while roots showed little accumulation (1.1 ± 0.23 mg kg^−1^). Translocation Factor (TF) and Bioaccumulation Factor (BAF) were calculated to be 13.64 and 0.06, respectively. Finally, As bioremediation potential of *Halomonas* strains and purified Exo1 was further analyzed directly under salt stress. [As(III)] was also estimated from roots and shoots of rice seedlings grown in normal pot soil containing indigenous As (2.96 mg kg^−1^ dry weight) derived from the environment. In control plants, roots and shoots contained 1.89 and 2 mg kg^−1^ of As, respectively and the TF and BAF were 1.06 and 0.64, respectively. 3.89 and 8.04 mg kg^−1^ of As were observed in the roots of plants treated with *Halomonas* sp. Exo1 and bacterial consortia, respectively. Shoots of plants treated with *Halomonas* sp. Exo1 and bacterial consortia contained 2.6 and 3.61 mg kg^−1^ of As, respectively. For plants treated with *Halomonas* sp. Exo1, the TF and BAF were 0.67 and 1.31, respectively. For plants treated with bacterial consortia, the TF and BAF were 0.45 and 2.72, respectively. Interestingly, for purified EPS-treated plants, the TF and BAF were 0.23 and 1.43, respectively.

## Discussion

Extracellular polymeric substances or exopolysaccharides (EPSs) are complex blend of high molecular weight microbial secretary biopolymeric by-products that are composed of organic macromolecules like polysaccharides along with smaller proportions of proteins, lipids, humic substances and uronic acids. Nevertheless, the fine structures of EPSs vary greatly among microbial genera and living habitats (Gupta and Diwan, 2017). Structural and compositional makeover of EPS favors the sequestration of metal ions that occurs through biosorption, by interaction between positively charged metal ions and negatively charged EPS. Abundant active and ionisable functional groups and non-carbohydrate substituents like acetamido, amine, sulfhydryl and carboxyl groups in proteins and phosphodiester (techoic acid), phosphate, hydroxyl groups in polysaccharides impart an overall negative charge to the biopolymer (Costa et al., 2018).

This study demonstrated the ability of *Halomonas* sp. Exo1 and its secreted EPS to sequester As confirming EPS-mediated As adsorption. However, there are reports of As-induced bacterial biofilm formation apart from As inducible EPS production, which can effectively adsorp the metalloid (Marchal et al., 2010, 2011). This study observed that salt acted as inducer of EPS production while addition of As in the salt-amended media enhanced EPS production. Role of salt in biofilm formation and/or EPS accumulation was already reported in a few halo-tolerant strains (Ozturk and Aslim, 2010; Qurashi and Sabri, 2012). Although production of EPSs in response to salt, starvation, dehydration, antibiotic or heavy metal stress had been reported (Gupta and Diwan, 2017), the role of EPS in osmoregulation was s least studied in general. Interestingly, the resistivity toward [As(III)] was also related to salt stress while in the absence of salt, the observed MIC value of *Halomonas* sp. Exo1 toward [As(III)] was lower than that found in the presence of salt. On the other hand, for [As(V)] no change in MIC values was noticed either in the presence or absence of salt. It is believed that some kind of positive feedback is operating in *Halomonas* sp. Exo1 to keep As mediated damage low. Increased As in the medium triggers the cells to synthesize more EPS, which sequesters more As thereby reducing the toxic effect of the metal.

In addition to the EPS-mediated As adsorption, *Halomonas* sp. Exo1 can bioremediate or detoxify As by oxidation of more toxic [As(III)] to less toxic [As(V)]. Apart from the biosorption of As, increase in MIC level of selected other HMs (Cr, Cd, and Mn) in presence of EPS indicated that EPS may have role in biosorption of these HMs too. Nevertheless, As resistant microbes employ different strategies to cope with As stress like oxidation, reduction, dissimilatory arsenate reducing activity and bioaccumulation through complexion, chelation etc. (Roy et al., 2015). Purified EPS was readily soluble in water and was produced maximally under metal (As) contaminated saline ambience during late stationary phase of bacterial growth. XRD data indicated that the EPS was amorphous in nature, which was further corroborated by the SEM analysis. EDX data showed that the EPS contains elements such as Na and Ca, which might aid in adsorption of oppositely charged metals like As from the ambient media and the sulfate group present in it confered its anionic character in the saline environment (Nielsen and Jahn, 1999). The distribution of cations such as Na^+^ and Ca^+^ in the EPS also suggested their ability to bind to the negative charges of the sulfate groups rendering them more bioavailable to plants. The present study shows the As bioadsorption potential of EPS both *in vivo* and *in vitro* in dose-dependent and time-dependent manner. It has been observed that with a linear increase in As dose, a proportional increase of the amount of EPS-mediated As bioadsorption occurred. However, with increase in time, the amount of bioadsorped As attained a steady state probably owing to the saturation of As binding sites in the EPS. In an earlier report, Deschatre et al. (2013) showed that one marine bacterium produced EPS that bioadsorped positively charged metal ions (Ag^+^ and Cu^2+^). Although, EPS-mediated HM sequestration was studied by several research groups (Pereira et al., 2011), the current study has its own merits in the sense that *in vivo* EPS-mediated metal sequestration is more physiologically relevant compared to the *in vitro* studies conducted by earlier groups.

Role of halo-tolerant PGPR in promoting growth of agricultural crops in soil suffering from increased salinity has been studied previously while only a few studies described the role of EPS on direct plant growth promotion. Most scientists reported that EPS promotes growth by its biocontrol property (Upadhyay et al., 2017). The present study focused on all the aspects of plant growth promotion and showed that application of pure EPS to the pot soil improved rice growth under abiotic stresses. The PGP traits of the whole cells under different conditions of salt and As stresses were investigated as these factors modulated EPS production. Halo-tolerant PGPR withstand high salinity by virtue of their efficient osmoregulatory mechanism that facilitates in carrying out normal cell functions in salt stress and they help salt-sensitive plants to overcome saline stress via restoration of their improper hormonal balance with activities that includes synthesis of essential phytohormones like indole-3-acetic acid (IAA), gibberellins (GA), cytokinins (CK), abscisic acid (ABA) together with solubilization of inorganic phosphate, nitrogen fixation and synthesis and excretion of essential metabolites such as siderophores and EPSs. The halo-rhizobacterial isolate under investigation exhibited PGP traits like IAA, NH_3_, HCN and siderophore production and inorganic phosphate solubilization. A preliminary investigation on N_2_-fixation property in the test organism indicated that the strain may be N_2_-fixer as it showed good growth in semi-solid Burk's N-free media. However, As exerted negative impact on all the PGP traits except production of siderophores, which increased with increase in As stress up to 6 mM. Siderophores are high-affinity, metal-binding plant metabolites secreted outside of the cell envelope. Present literature suggests that siderophores that are produced under conditions of iron (Fe) deficiency to quench Fe from the surroundings are also able to effectively bind other metal and metalloids including As (Rajkumar et al., 2010). Siderophores also take part in preventing infections from several phytopathogens such as *Fusarium oxysporium* by making Fe less available to certain pathogenic microbes, and therefore act as an essential biocontrol metabolite (Berthelin et al., 1991). As this strain also showed antagonism against *Fusarium oxysporium*, so in addition to the direct growth-promoting features of the PGPR in this study, *Halomonas* sp. Exo1 could be effective as biocontrol agent thereby boosting plant growth indirectly by keeping away phytopathogenic bacteria and fungi. PGP activities of *Halomonas* and other halo-tolerant bacteria that work under salt and HM stresses have already been reported from a few studies (Siddikee et al., 2010; Desale et al., 2014). Moreover, some of them are EPS producers but none of the studies revealed the role of EPS with direct plant growth promotion. This is the first report where EPS was found to directly enhance rice growth. Nevertheless, Arora et al. (2010) showed that crude cyanobacterial EPS from non-halophilic *Nostoc* species enhanced seed germination and growth vigor of rice, wheat and maize (plate-based assay) by binding the free Na^+^ ions from aqueous media. They also hypothesized the possible role of EPS in increasing the growth vigor of the seedlings. Interestingly, the present study demonstrated the ability of *Halomonas* sp. Exo1 to bioaccumulate essential osmolytes including Na^+^ and K^+^, and showed direct *in vitro* evidence for EPS-mediated sequestration of Na^+^ ions. Presence of increased level of Na^+^ in NaCl-treated EPS was further corroborated by SEM-EDX analysis. Therefore, consistent with the results of Arora et al. (2010), one of the mechanisms of plant growth promotion by purified EPS (Exo1) is through salt stress alleviation by EPS-mediated Na^+^ ion chelation and making them less available to plants under saline conditions. Moreover, EPS can improve soil structure by binding to soil particles to form microaggregates and macroaggregates and improves the water holding capacity of soil (Amellal et al., 1998). EPS produced by *Paenibacillus polymyxa* increases the aggregation of root adhering soil/root tissue ratio in wheat (Amellal et al., 1998). Additionally, plants that harbor EPS-producing rhizobacteria have selective advantage over others during environmental stresses like excessive salt and water logging. Yi et al. (2008) suggested the role of EPSs in inorganic phosphate solubilization and plant growth promotion. However, the actual mechanism by which bacteria employ EPS in inorganic phosphate solubilization is yet to be elucidated. Another role of EPS is antioxidant activity (Kohler et al., 2009), which has also been reflected in the present study where EPS derived from *Halomonas* sp. Exo1 showed DPPH-free radical scavenging activity.

Most of the rice varieties including Jarava used in this study are non-tolerant varieties that do not survive at higher concentration of As. Nevertheless, As translocation was lower in the microbe-treated plants than the control plants. Hence the application of As-accumulating rhizomicrobes decreases the risk of As food chain contamination. Another advantage of the test rhizobacterium, used as bioinoculum, is that it does not exhibit antibiotic resistance properties and hence is absolutely fit for field applications (data not shown). Moreover, antifungal property and presence of siderophores would help the plants to survive in the competitive environment of the rhizosphere.

Therefore, the isolated salt- and As-tolerant PGPR and its EPS would be ideal candidates for application as biofertilizers or as bioinoculants, which would help in bioremediation of contaminated saline soil and support agronomy of salt-tolerant rice cultivation through salinity stress alleviation.

## Conclusion and Future Outlook

This research work demonstrated that the salt and As-tolerant strain *Halomonas* sp. Exo1 facilitated growth promotion of a salt-tolerant rice variety in the presence of growth inhibitory level of salt. Growth promotion of rice by the *Halomonas* strain under salt and As stresses occurred primarily through its PGP traits (production of IAA, siderophores, NH_3_ and HCN, Pi solubilization, non-symbiotic N_2_ fixation and anti-microbial or biocontrol activities) and most importantly through EPS secretion. The *Halomonas* sp. Exo1 was found capable of accumulating high amount of As within its cell-biomass. Although, the exact location of accumulation of As within the cell could not be exclusively ascertained in this study but *in vitro* EPS mediated and dead cell biomass-mediated [As(III)] biosorption studies supported the hypothesis that majority of As sequestration in *Halomonas* sp. Exo1 occurred through EPS-mediated bioadsorption. Among the other functional roles of the purified EPS, *in vitro* Na^+^ ion sequestration and antioxidant activity were significant. Finally, direct growth-promoting effect of the purified EPS was observed based on the increased growth vigor of the salt-tolerant rice seedlings in pot-based bioassay. Therefore, this study established the role of halo-rhizobacterial EPS in promoting plant growth where EPS-mediated growth promotion of rice perhaps occurred through salt stress alleviation, inorganic phosphate solubilization and enhanced nutrient uptake. So, the overall results of the present research suggested that the isolated halo-PGPR and its purified EPS would find application as bioinoculant or biofertilizer in the cultivation of salt-tolerant crops such as rice in the low-lying contaminated coastal areas.

## Author Contributions

AM provided the soil and plant samples and helped in outsourcing. PM carried out all the lab based experiments with guidance from MR. MR and PM prepared the manuscript. The manuscript was checked several times by the three authors and all of them agreed on the final version.

### Conflict of Interest Statement

The authors declare that the research was conducted in the absence of any commercial or financial relationships that could be construed as a potential conflict of interest.
