# Supplementary material for: Halomonas Rhizobacteria of Avicennia marina of Indian Sundarbans Promote Rice Growth Under Saline and Heavy Metal Stresses Through Exopolysaccharide Production
Source: Front Microbiol. 2019 May 29;10:1207. doi: 10.3389/fmicb.2019.01207 (PMC6549542; doi:10.3389/fmicb.2019.01207)
Supplement: Supplementary file 1 [file Table_1.DOCX]

**Supplementary Table S1**

Physico-chemical properties of soil from *Avicennia marina* rhizosphere including biologically available heavy metal level

| **Heavy metals** | **Values** | **Other Properties** | **Values** |
| --- | --- | --- | --- |
| Cu (ppm) | 21.88 | pH | 7 |
| Mn (ppm) | 30.23 | Total organic matter (%) | 0.64 |
| Fe (ppm) | 62.04 | Soil Organic Carbon (%) | 0.48 |
| Co (ppm) | 2.02 | Moisture content (%) | 5 – 6 |
| Ni (ppm) | 3.53 | EC (mS/cm) | 1.70 |
| Pb (ppm) | 1.85 | Available N (mg kg^-1^) | 67.91 |
| Cd (ppm) | 1.08 | Available K (mg kg^-1^) | 7978.65 |
| Cr (ppm) | 0.782 | Available P (mg kg^-1^) | 56.99 |
| Zn (ppm) | 39.85 |  |  |
| Mo(ppm) | BDL* |  |  |

*BDL means “Below Detectable Level”.
