# Supplementary material for: Halomonas Rhizobacteria of Avicennia marina of Indian Sundarbans Promote Rice Growth Under Saline and Heavy Metal Stresses Through Exopolysaccharide Production
Source: Front Microbiol. 2019 May 29;10:1207. doi: 10.3389/fmicb.2019.01207 (PMC6549542; doi:10.3389/fmicb.2019.01207)
Supplement: Supplementary file 2 [file Table_2.docx]

**Supplementary Table S2**

Effect of salt stress on elemental composition of EPS (Exo1)

| **Elements** | **Standard** | **Crude EPS Exo1** | | **Purified EPS Exo1 (Control)** | | **Purified EPS Exo1 + 5% NaCl** | | **Purified EPS Exo1 + 15% NaCl** | |
| --- | --- | --- | --- | --- | --- | --- | --- | --- | --- |
|  |  | **Weight %** | **Atomic %** | **Weight %** | **Atomic %** | **Weight %** | **Atomic %** | **Weight %** | **Atomic %** |
| **C** | **CaCO_3_** | 8.84 | 14.79 | 52.60 | 59.77 | 49.35 | 56.97 | 50.04 | 59.18 |
| **N** | **Not defined** | 3.26 | 4.68 | 15.55 | 15.15 | 14.60 | 14.46 | 11.11 | 11.27 |
| **O** | **SiO_2_** | 40.52 | 50.89 | 26.11 | 22.27 | 28.99 | 25.12 | 25.92 | 23.01 |
| **P** | **GaP** | 0.42 | 0.27 | 0.15 | 0.07 | 0.26 | 0.12 | 0.04 | 0.02 |
| **S** | **FeS_2_** | 5.81 | 3.64 | 0.51 | 0.22 | 0.77 | 0.33 | 0.54 | 0.24 |
| **Na** | **Albite** | 15.10 | 13.20 | 1.13 | 0.67 | 2.95 | 1.78 | 3.19 | 1.97 |
| **Cl** | **KCl** | 14.81 | 8.40 | 0.85 | 0.33 | 2.68 | 1.05 | 3.38 | 1.35 |
| **K** | **MAD-10 Feldspar** | 0.51 | 0.26 | 0.07 | 0.02 | 0.14 | 0.05 | 0.11 | 0.04 |
| **Mg** | **MgO** | 2.02 | 1.67 | 0.02 | 0.01 | 0.16 | 0.09 | 0.12 | 0.07 |
| **Ca** | **Wollastonite** | 1.02 | 0.51 | 0.19 | 0.07 | 0.11 | 0.04 | 0.23 | 0.08 |
| **Fe** | **Fe** | 0.06 | 0.02 | 0.0 | 0.0 | 0.0 | 0.0 | 0.0 | 0.0 |
| **Cu** | **Cu** | 0.10 | 0.03 | 0.0 | 0.0 | 0.0 | 0.0 | 0.0 | 0.0 |
| **Zn** | **Zn** | 0.45 | 0.14 | 0.0 | 0.0 | 0.0 | 0.0 | 0.0 | 0.0 |
| **Mo** | **Mo** | 6.83 | 1.43 | 0.0 | 0.0 | 0.0 | 0.0 | 0.0 | 0.0 |
| **Cr** | **Cr** | 0.11 | 0.04 | 0.0 | 0.0 | 0.0 | 0.0 | 0.0 | 0.0 |
| **As** | **InAs** | 0.13 | 0.04 | 0.0 | 0.0 | 0.0 | 0.0 | 0.0 | 0.0 |
| **Al** | **Al_2_O_3_** | 0.0 | 0.0 | 2.81 | 1.42 | 0.0 | 0.0 | 3.86 | 2.03 |
| **Si** | **SiO_2_** | 0.0 | 0.0 | 0.0 | 0.0 | 0.0 | 0.0 | 1.46 | 0.74 |
| **Totals** | | 100.00 | | | | | | | |

No peaks were omitted; Number of iterations = 7
