# Supplementary material for: Halomonas Rhizobacteria of Avicennia marina of Indian Sundarbans Promote Rice Growth Under Saline and Heavy Metal Stresses Through Exopolysaccharide Production
Source: Front Microbiol. 2019 May 29;10:1207. doi: 10.3389/fmicb.2019.01207 (PMC6549542; doi:10.3389/fmicb.2019.01207)
Supplement: Supplementary file 3 [file Data_Sheet_1.docx]

**Supplementary File**

**RESULTS**


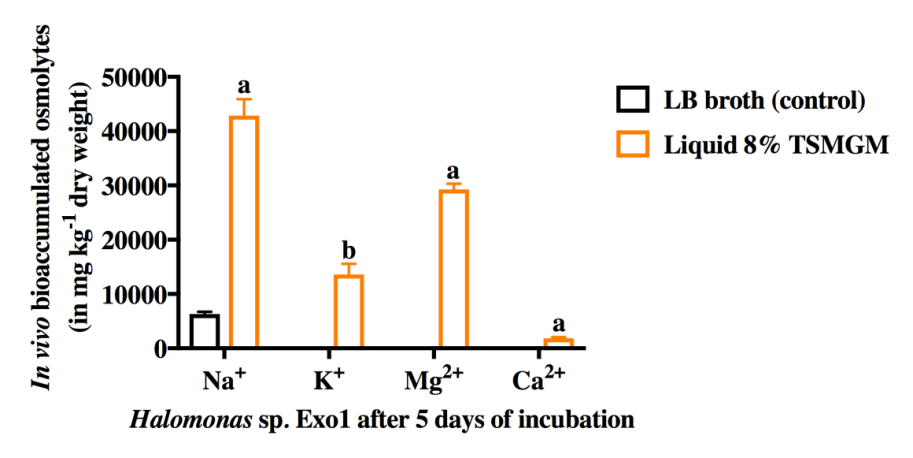


**SUPPLEMENTARY FIGURE S1 *In vivo* bioaccumulation of osmolytes in *Halomonas* sp. Exo1. The amounts of various osmolytes (Na^+^, K^+^, Mg^2+^ and Ca^2+^) accumulated within the cell-biomass of *Halomonas* sp. Exo1 grown for 5 days in LB broth (control) and liquid 8% TSMGM.**


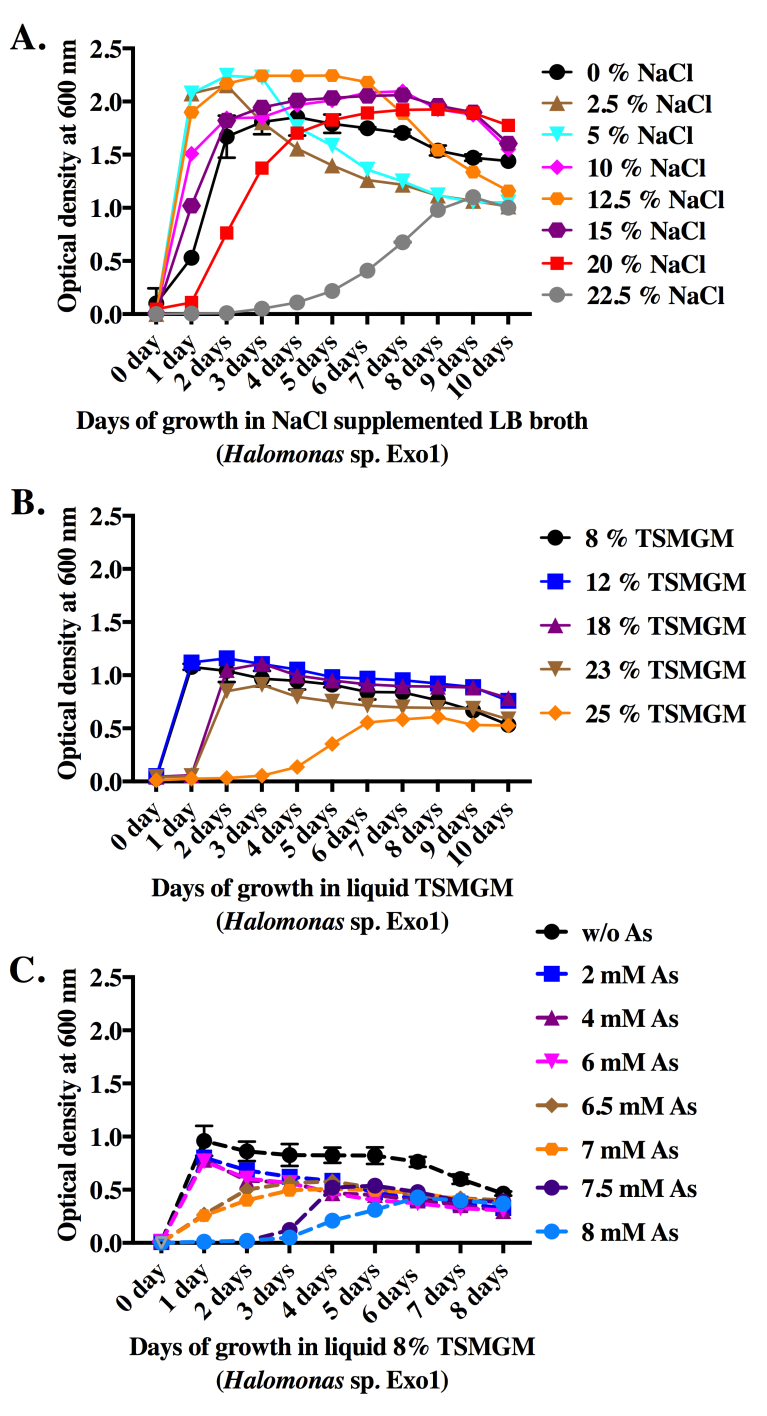


**SUPPLEMENTARY FIGURE S2 Salt tolerance and growth profile of one selected strain *Halomonas* sp. Exo1 under salt and arsenic stresses. Growth kinetics of *Halomonas* sp*.* Exo1 in LB broth with increasing concentration of NaCl is shown in (A), and in liquid TSMGM with increasing concentration of total salts is shown in (B). 100 ml of 8%, 12%, 18%, 23% and 25% TSMGM contains 6.4% (1.1 M), 9.6% (1.64 M), 14.37% (2.46 M), 18.46% (3.16 M) and 20% (3.42 M) NaCl respectively. (C). Growth kinetics of *Halomonas* sp*.* Exo1 in liquid 8% TSMGM in presence and absence of [As(III)].**


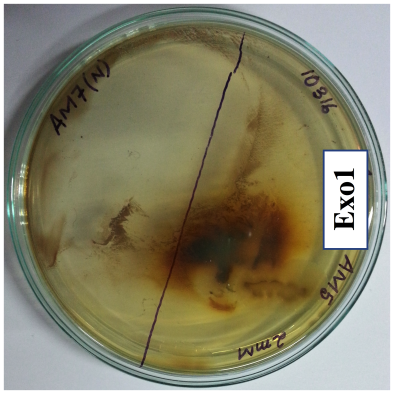


**SUPPLEMENTARY FIGURE S3 Ag(NO)_3_ plate assay for arsenic biotransformation. Representative image of plate containing halo-rhizobacterial spot inoculant (*Halomonas* sp. Exo1) sprayed with AgNO_3_ showing distinct brownish-red precipitate around the spot inoculant. Absence of light yellow precipitate (indicative of lack of As reduction) confirmed As oxidation.**


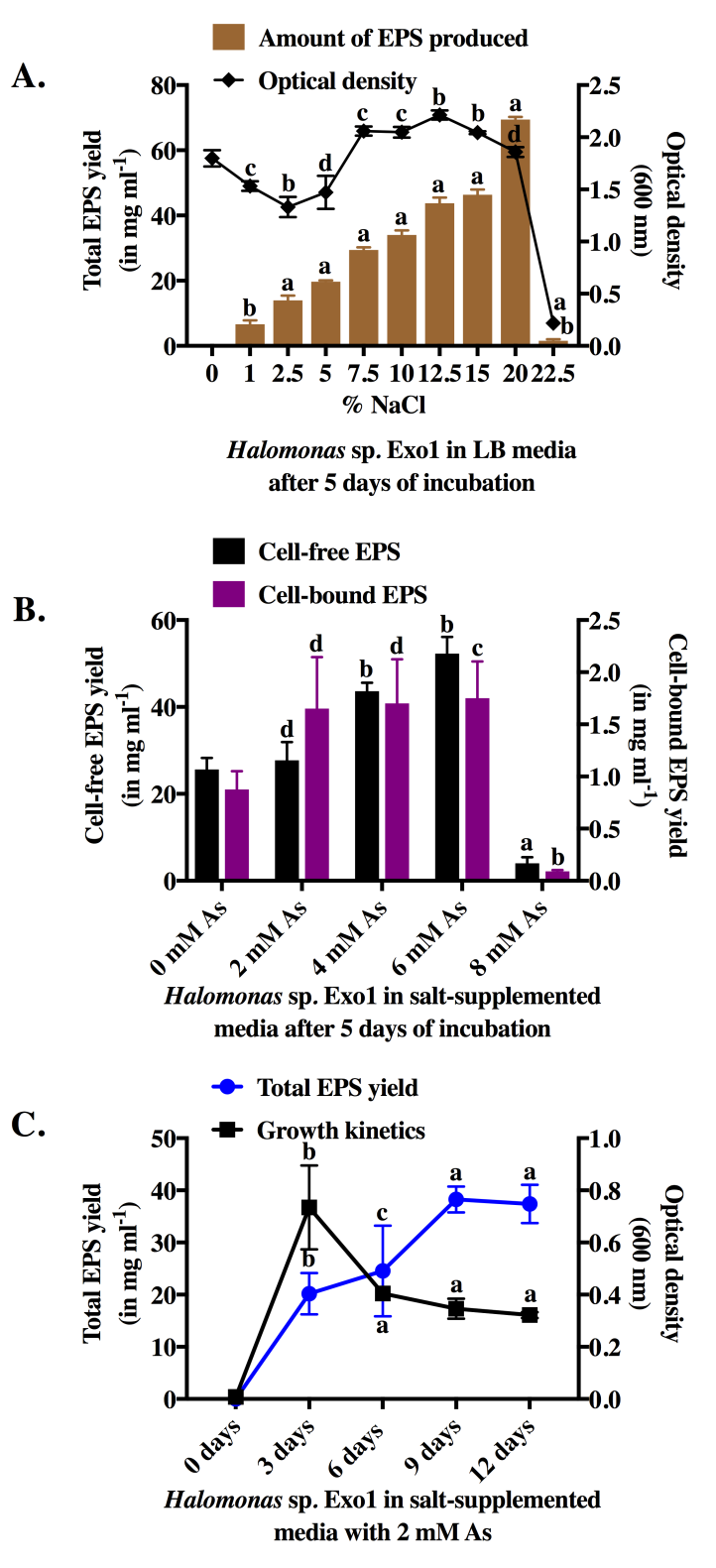


**SUPPLEMENTARY FIGURE S4 Effects of salt and arsenic on EPS yield. (A) EPS production (in context to optical density) in LB media with increasing NaCl concentrations by one selected isolate *Halomonas* sp. Exo1. (B) Production of cell-free and cell-bound EPS in 8% TSMGM with increasing concentrations of [As(III)] (2, 4, 6 mM). (C) Comparison of growth kinetics with total EPS yield over time of *Halomonas* sp. Exo1 in 8% TSMGM containing 2 mM [As(III)]. Error bars represent mean ± standard deviation of triplicate values.**


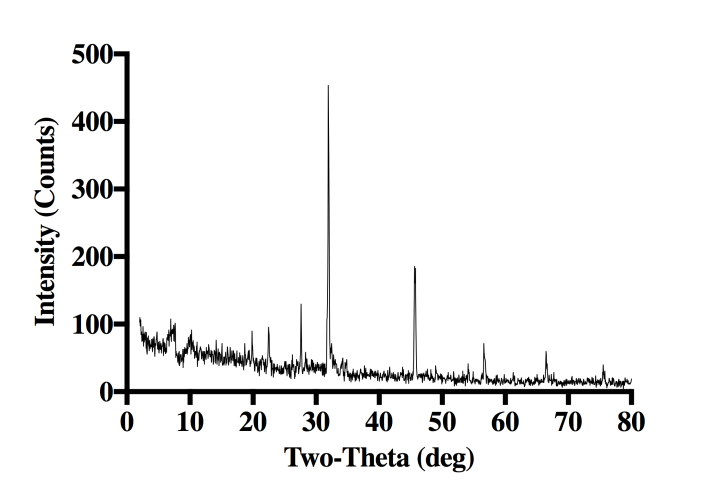


**SUPPLEMENTARY FIGURE S5 XRD profile of cell-free EPS derived from *Halomonas* sp*.* Exo1.**


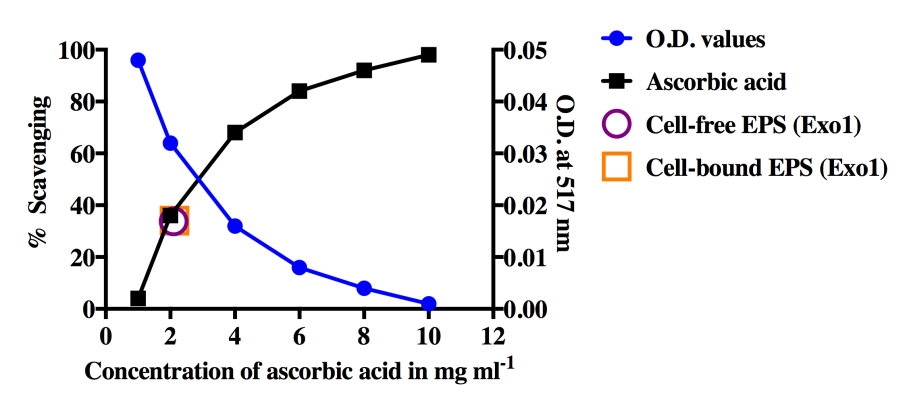


**SUPPLEMENTARY FIGURE S6 Antioxidant activity of EPS (Exo1). % DPPH-free radical scavenging assay of cell-free and cell-bound EPSs derived from *Halomonas* sp. Exo1 with respect to known ascorbic acid standard.**


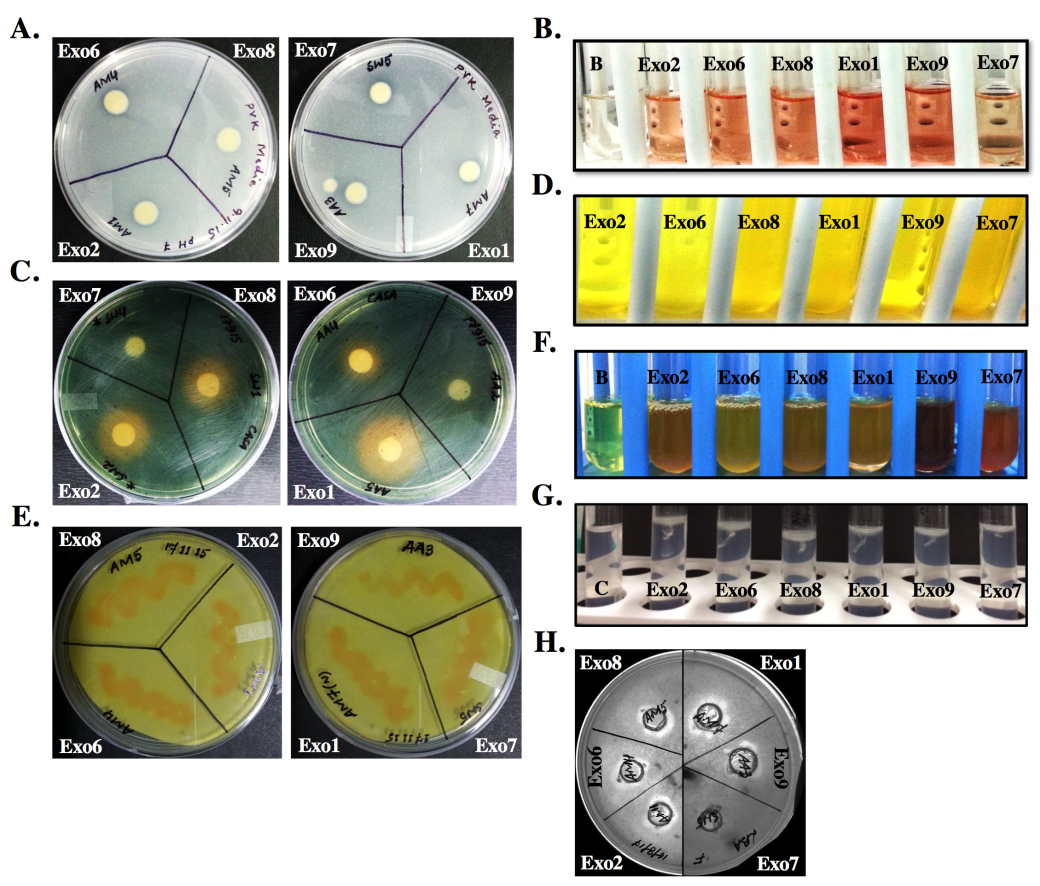


**SUPPLEMENTARY FIGURE S7 Representative images of *in vitro* PGP traits as displayed by the 6 halo-rhizobacterial isolates. (A) NBRIP media plates showing zone of phosphate solubilization around the spot inoculants. (B) IAA production. (C) Siderophore production. (D) NH_3_ production. (E) HCN production in plate-based assay. (F) HCN production in broth-based assay. (G) Growth of *Halomonas* strains in Burk’s N-free media (with saline concentration of 7.5% w/v) after 7 days at 32°C. (H) Zone of inhibition of *Fusarium oxysporium* created by the *Halomonas* strains showing anti-fungal activity. ‘B’ and ‘C’ stand for blank and control respectively.**


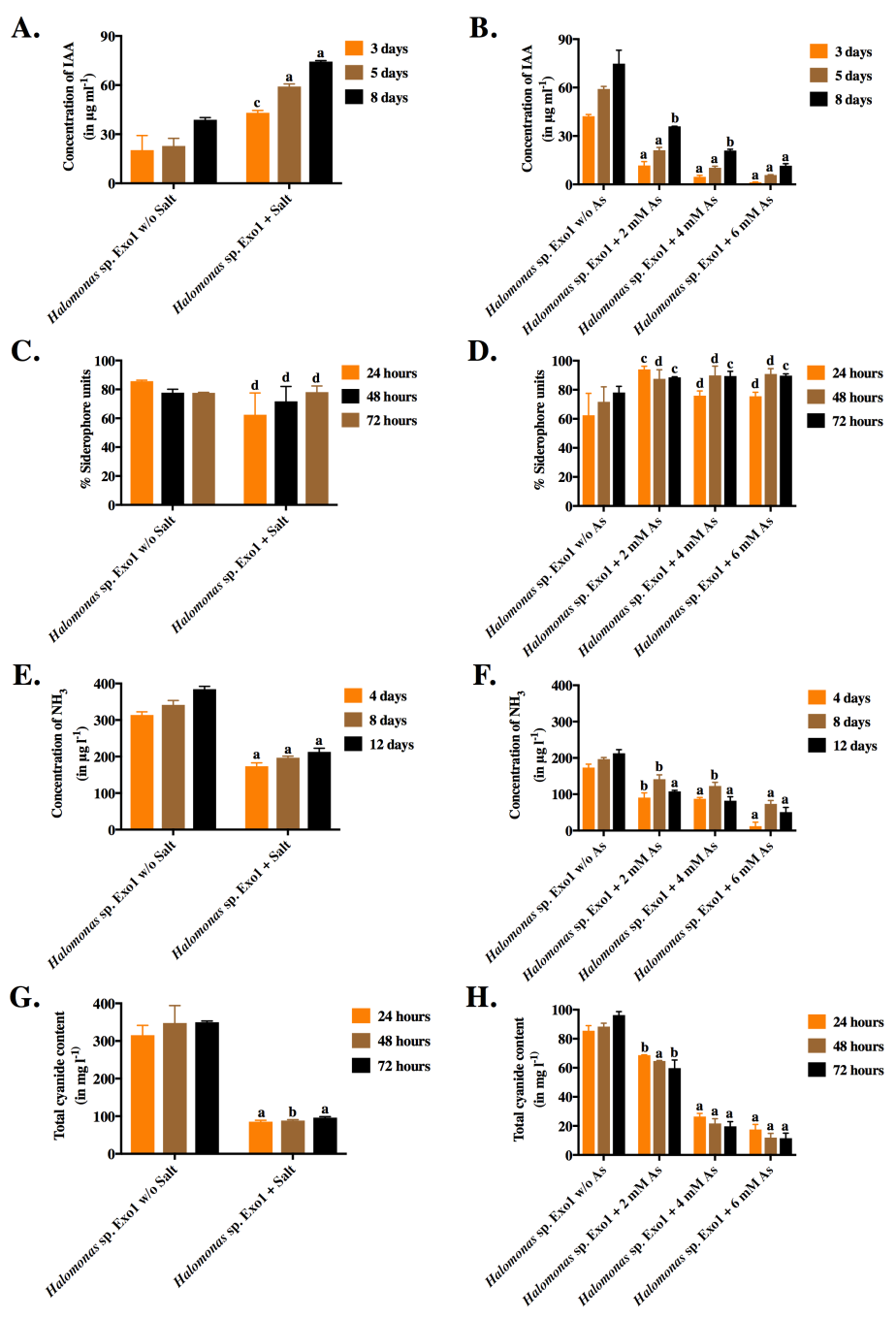


**SUPPLEMENTARY FIGURE S8. Production of essential plant growth-promoting metabolites by *Halomonas* sp. Exo1. Indole-3-acetic acid (IAA) production (A-B). (A) In salt-deficient (LB) and salt-supplemented (8% TSMGM) media. (B) In salt-supplemented (8% TSMGM) media in absence and presence of As (2, 4, 6 mM). Concentration of L-Trp used is 500 mg l^-1^ in all three cases. Siderophore production (C-D). (C) In salt-deficient (CAS + King’s B + 1% NaCl) and salt-supplemented (CAS + King’s B + 5% NaCl) media. (D) In salt-supplemented (CAS + King’s B + 5% NaCl) media in absence and presence of As (2, 4, 6 mM). Ammonia (NH_3_) production (E-F). (E) In salt-deficient (peptone water) and salt-supplemented (peptone water) media. (F) In salt-supplemented (peptone water) media in absence and presence of As (2, 4, 6 mM). HCN production (G-H). (G) In salt-deficient (LB) and salt-supplemented (8% TSMGM) media. (H) In salt-supplemented (8% TSMGM) media in absence and presence of As (2, 4, 6 mM). Error bars represent mean ± standard deviation of triplicate values.**

**
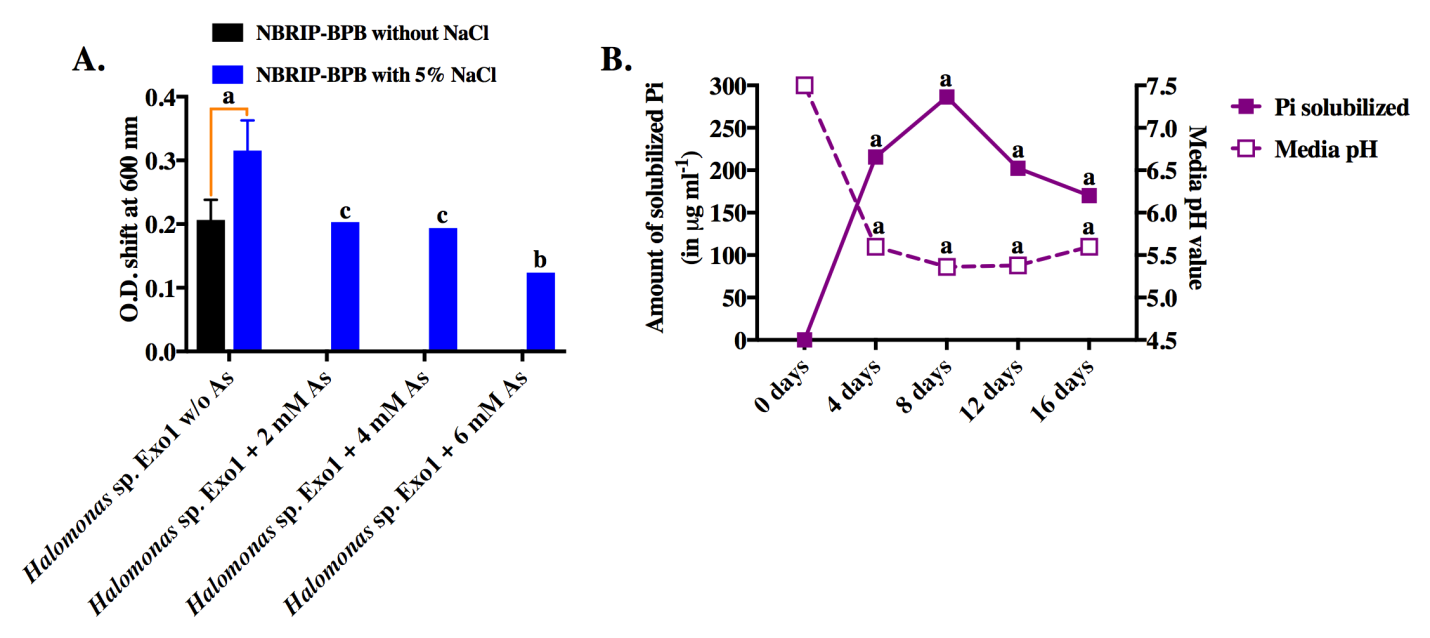
SUPPLEMENTARY FIGURE S9 Inorganic phosphate (Pi) solubilization and organic acid production. (A). Shift in optical density (A_600_) in salt-deficient and salt-supplemented NBRIP-BPB media in absence and presence of [As(III)] (2, 4, 6 mM) stress of *Halomonas* sp. Exo1. (B) Amount of Pi solubilized over time (up to 16 days of bacterial growth in NBRIP media with initial pH 7.5 during inoculation) by *Halomonas* sp. Exo1 (bold lines, closed symbols) and concomitant drop in media pH value (dotted lines, open symbols).**


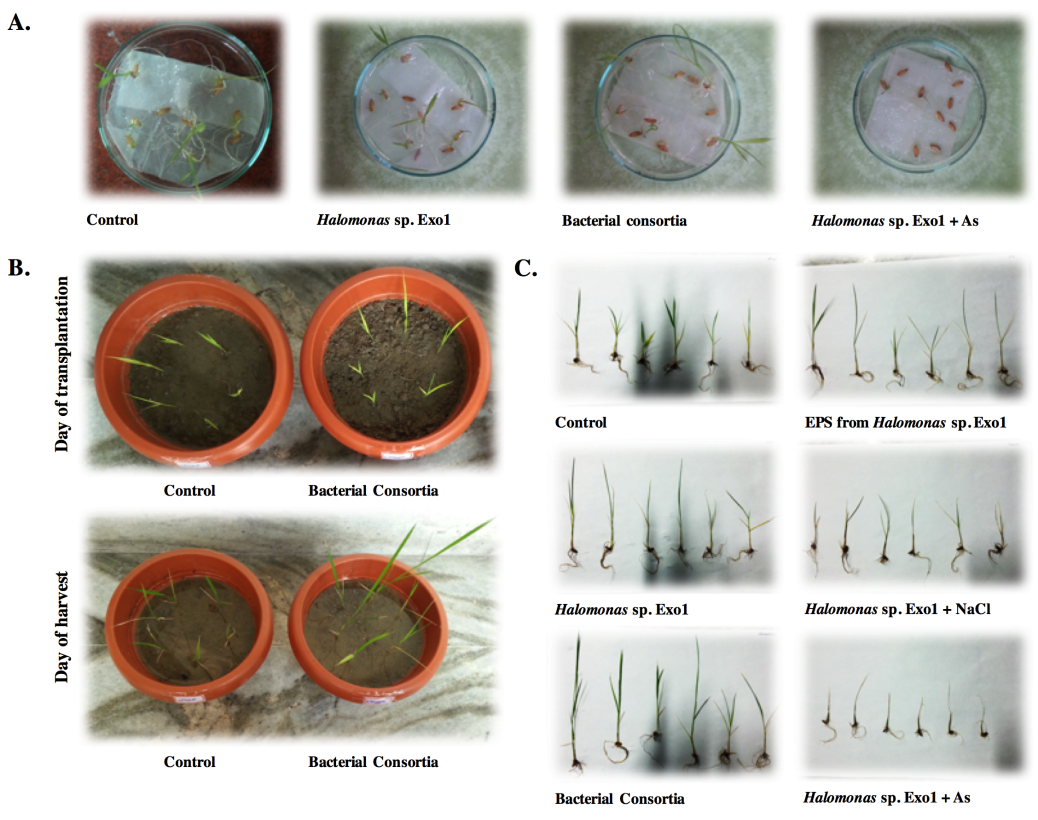


**SUPPLEMENTARY FIGURE S10 Pot-based growth assay with salt-tolerant rice seedlings and halo-PGPRs. (A) Representative images of plate-based germination assay on 7^th^ day of germination of control and treated seeds (B) Representative images of pot-based growth assay [plantlets inoculated with dH_2_O (left) and with bacterial consortia (right)] showing growth of rice seedlings on the day of transplantation (i.e., 15^th^ day, above) and day of harvest (i.e., 30^th^ day, below). (C) Images of post-harvest rice seedlings (30^th^ day) from pots with various treatment conditions.**
